# Jasmonic acid is a downstream component in the modulation of somatic embryogenesis by Arabidopsis Class 2 phytoglobin

**DOI:** 10.1093/jxb/erw022

**Published:** 2016-03-09

**Authors:** Mohamed M. Mira, Owen S. D. Wally, Mohamed Elhiti, Adel El-Shanshory, Dhadi S. Reddy, Robert D. Hill, Claudio Stasolla

**Affiliations:** Department of Plant Science, University of Manitoba, Winnipeg, Manitoba, R3T 2N2, Canada

**Keywords:** Auxin, PGB2, jasmonic acid, nitric oxide, phytoglobin, somatic embryogenesis.

## Abstract

Previous studies have shown that the beneficial effect of suppression of the Arabidopsis phytoglobin 2 gene, *PGB2*, on somatic embryogenesis occurs through the accumulation of nitric oxide (NO) within the embryogenic cells originating from the cultured explant. NO activates the expression of *Allene oxide synthase* (*AOS*) and *Lipoxygenase 2* (*LOX2*), genes encoding two key enzymes of the jasmonic acid (JA) biosynthetic pathway, elevating JA content within the embryogenic tissue. The number of embryos in the single *aos1-1* mutant and *pgb2-aos1-1* double mutant declined, and was not rescued by increasing levels of NO stimulating embryogenesis in wild-type tissue. NO also influenced JA responses by up-regulating *PLANT DEFENSIN 1* (*PDF1*) and *JASMONATE-ZIM-PROTEIN* (*JAZ1*), as well as down-regulating *MYC2.* The NO and JA modulation of *MYC2* and *JAZ1* controlled embryogenesis. Ectopic expression of *JAZ1* or suppression of *MYC2* promoted the formation of somatic embryos, while repression of *JAZ1* and up-regulation of *MYC2* reduced the embryogenic performance. Sustained expression of *JAZ1* induced the transcription of several indole acetic acid (IAA) biosynthetic genes, resulting in higher IAA levels in the embryogenic cells. Collectively these data fit a model integrating JA in the PGB2 regulation of Arabidopsis embryogenesis. Suppression of *PGB2* increases JA through NO. Elevated levels of JA repress *MYC2* and induce *JAZ1*, favoring the accumulation of IAA in the explants and the subsequent production of somatic embryos.

## Highlight

Suppression of the phytoglobin GLB2 enhances jasmonic acid which promotes the accumulation of auxin and the formation of somatic embryos in Arabidopsis

## Introduction

Found in all nucleated organisms, hemoglobins are important Fe heme-containing proteins fulfilling a variety of tasks. Initially characterized in vertebrates in relation to their ability to bind and transport oxygen and other ligands such as CO_2_, and NO, hemoglobin-like compounds were also found expressed in the nodules of legumes containing nitrogen-fixing bacteria where they served to prevent inactivation of nitrogenase by binding oxygen (reviewed in Smagghe *et al.*, 2009). Additional plant hemoglobin-like coumpounds were subsequently found to be more widely distributed in plants (Hill, 2012) and were ascribed the name, nonsymbiotic hemoglobins to distinguish them from leghemoglobin. The types (Garrocho-Villegas *et al.*, 2007) and function (Hill, 2014) of nonsymbiotic hemoglobins have expanded considerably to the point that a more specific name, phytoglobin (Pgb), has been introduced.

From a phylogenetic perspective, oxygen binding activity, and the expression profile, three types of plant Pgbs have been described (Hunt *et al.*, 2001). Class 1 and 2 Pgbs possess a 3-on-3 α-helical loop surrounding the heme moiety, while class 3 Pgbs are similar to truncated bacterial globins (Watts *et al.*, 2001). The majority of the studies are centered on class 1 and 2 which are characterized by a very high oxygen binding affinity: an approximate *K*_m_ of 2nM for members of class 1 and 150nM for members of class 2 (Hoy and Hargrove, 2008; Dordas, 2009). The high affinity for oxygen exhibited by class 1 Pgbs discounts their roles in oxygen sensing and transport (Hill, 1998). The most plausible function of plant Pgbs is to scavenge NO, as demonstrated in several developmental and stress responses (reviewed in Hill, 2012).

Three *Pgb* genes categorized in their respective classes have been identified in Arabidopsis: *PGB1*, *PGB2*, and *PGB3*. While no information is available on the function of *PGB3*, several studies have provided evidence for the role of *PGB1* and *PGB2* during hypoxia. Due to its ability to scavenge NO efficiently under hypoxic conditions, PGB1 exercises a protective role during abiotic stress (Perazzolli *et al.*, 2004). Arabidopsis roots grown under low oxygen conditions rapidly expressed *PGB1*, and plants overexpressing *PGB1* exhibited a higher survival rate resulting from a depletion in cellular NO (Hunt *et al.*, 2002). The role of protection from hypoxia observed for PGB1 was also documented with other class 1 Pgbs. Hypoxic roots of alfalfa overexpressing a class 1 *Pgb* developed fewer aerenchyma and exhibited enhanced growth due to sustained NO-scavenging mechanisms (Dordas *et al.*, 2003). Opposite results were observed in plants suppressing the barley *Pgb* (Dordas *et al.*, 2003).

Like *PGB1*, overexpression of *PGB2* increased NO scavenging (Hebelstrup and Jensen, 2008; Hebelstrup *et al.*, 2012) and enhanced plant survival under hypoxic conditions (Hebelstrup *et al.*, 2006). Expression of *PGB2* is favored by cytokinin and low temperatures (Trevaskis *et al.*, 1997; Hunt *et al.*, 2001), conditions not affecting *PGB1* and denoting differential control mechanisms and possibly functions of the two Pgbs. The preferential expression of *PGB2* in immature and developing organs, such as somatic embryos, leaflets, and immature seeds and fruits (Hendriks *et al.*, 1998; Hunt *et al.*, 2002; Wang *et al.*, 2003), is suggestive of a function for the gene to accommodate the high energy demand of the organs. In developing Arabidopsis seeds, overexpression of *PGB2* has been associated with improved oil accumulation through the maintenance of a high energy status (Vigeolas *et al.*, 2011).

A recurring theme emerging from these studies is the mediation of NO in many Pgb-regulated events. The prominent role of NO as a signal molecule in many physiological responses, in conjunction with the expression of Pgbs under normoxic conditions (Hill, 2012), suggests a possible involvement of Pgbs in fundamental developmental processes. Major phenotypic defects were observed in Arabidopsis plants with altered *PGB1* and *PGB2* expression (Hebelstrup *et al.*, 2006). Independent evidence suggests a control of meristem function by Pgbs. While overexpression of either *PGB1* or *PGB2* encourages the vegetative–reproductive transition of the shoot meristem, the repression of *PGB1* affects the time of flowering (Hebelstrup and Jensen, 2008). Meristem formation *in vitro* was also affected by Pgbs, with the overexpression of both *PGB1* and *PGB2* favoring the formation of shoots through the activation of auxin and cytokinin perception (Wang *et al.*, 2011). By modulating NO emission, *PGB1* and *PGB2* also regulate hyponastic responses during flooding, an observation integrating Pgbs in long-range plant signaling mechanisms (Hebelstrup *et al.*, 2013).

While the participation of Pgbs in developmental processes has mainly been investigated post-embryonically, Pgbs might play a central role during plant embryogenesis. Phytoglobin genes are expressed during embryo development (Smagghe *et al.*, 2007), and when hemoglobin is applied exogenously it influences somatic embryogenesis, the ability of somatic cells to produce embryos (Jayabalan *et al.*, 2004). A more direct involvement of Pgbs during embryo formation was demonstrated by Elhiti *et al.* (2013) using Arabidopsis somatic embryogenesis. Suppression of *PGB2* increased embryogenesis by elevating NO levels at the sites of the explants forming somatic embryos. Accumulation of NO suppresses MYC2 (Elhiti *et al.*, 2013), a basic helix–loop–helix (bHLH) domain-containing transcription factor which represses the biosynthesis of auxin (Dombrecht *et al.*, 2007), the inductive signal which initiates the embryogenic process (Raghavan, 2004). As a result of this mechanism, *PGB2*-suppressed cells accumulate more auxin and produce a large number of somatic embryos (Elhiti *et al.*, 2013). While representing a valid framework integrating Pgb signaling in plant embryogenesis, this model is most probably incomplete in terms of the number of intermediates transducing the PGB2 response. During post-embryonic growth, both NO and MYC2 operate at the interphase of a variety of transduction pathways often involving hormones, predominantly jasmonic acid (JA) (Chen *et al.*, 2011; Mur *et al.*, 2013). While the link between MYC2 and JA signaling has been well established during plant–pathogen interactions and insect predation (Lorenzo and Solano, 2005), the relationship between NO and JA is far from clear. JA synthesis is repressed by NO in some systems but induced in others (Orozco-Cardenas and Ryan, 2002). A rapid induction in JA level following inoculation with *Botryts cinerea* was observed in Arabidopsis plants accumulating NO through suppression of *Pgb* (Hebelstrup *et al.*, 2012). Based on these observations, it cannot be excluded that JA plays a key role during embryogenesis, possibly as an integrated component of the PGB2 regulatory mechanisms.

**Fig. 1.**
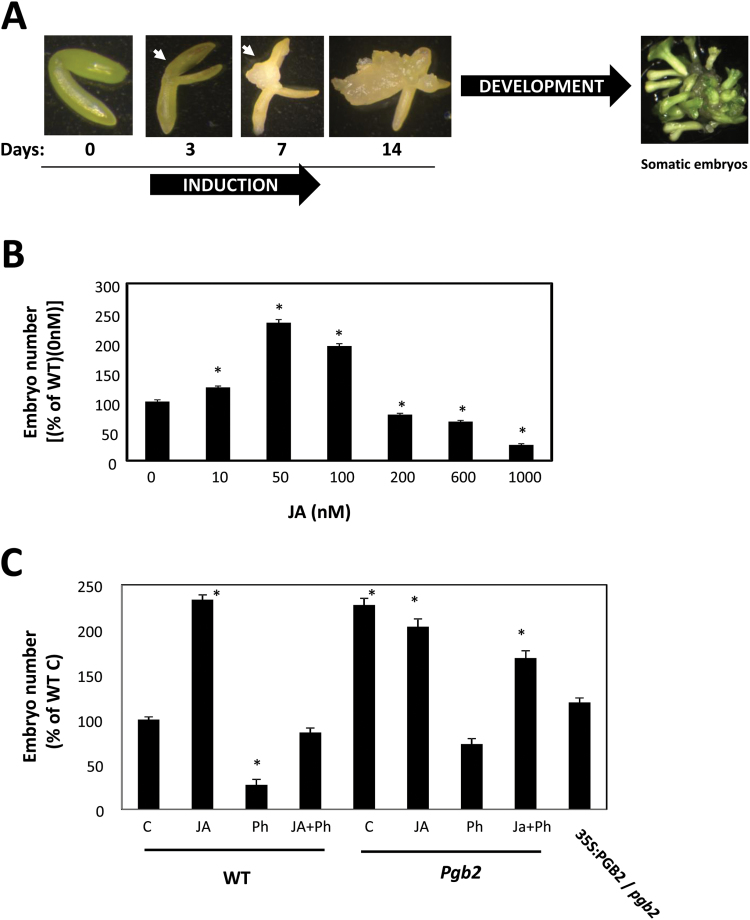
Effects of altered levels of jasmonic acid (JA) on Arabidopsis somatic embryogenesis. (A) Arabidopsis somatic embryos are generated through a two-step process. Dissected zygotic embryos are initially plated on 2,4-D-containing induction medium required for the formation of embryogenic tissue. After 14 d, the explants are transferred onto a hormone-free development medium which stimulates the production of somatic embryos. Fully developed somatic embryos can be obtained after 9 d on development medium. Arrows indicate the formation of the embryogenic tissue. (B) Changes in embryo number in the wild type (WT) line cultured with increasing levels of JA applied to the induction medium. Values are means ±SE of at least three biological replicates. An asterisk indicates statistically significant differences (*P*≤0.005) from the control (JA=0nM) value set at 100%. (C) Effects of applications of JA (50nM) and/or Phenidone (Ph) on the number of somatic embryos produced by the WT, *pgb2*, and 35S:PGB2/*pgb2* lines. Values are means ±SE of at least three biological replicates. An asterisk indicates statistically significant differences (*P*≤0.005) from value of the WT (C) set at 100%. (This figure is available in colour at *JXB* online.)

In an effort to establish a relationship between PGB2, NO, and JA in the regulation of embryo formation, we used the well-characterized Arabidopsis somatic embryogenesis system (Elhiti *et al.*, 2013). Formation of somatic embryos in Arabidopsis is a two-step process (Fig. 1A). The first involves culturing early cotyledonary zygotic embryos on an auxin-containing induction medium which stimulates the formation of embryogenic tissue. Production of somatic embryos from the embryogenic tissue is then initiated by the removal of auxin (Bassuner *et al.*, 2007). Our results suggest that JA is a key component of PGB2 regulation of embryogenesis in a model including NO and several JA-responsive intermediates.

## Materials and methods

### Plant materials

The Arabidopsis (Columbia) mutant lines *ami1-1* (SALK_069970) (Elhiti *et al.* 2013), *jaz1-1* (SALK 011957) (Demianski *et al.*, 2012), and *aos1-1* (SALK 017756) (Park *et al.*, 2002), and the *pASA1:GUS* reporter line (CS16701), were obtained from the Arabidopsis Biological Resource Center (ABRC). The following lines were received as gifts: the *pgb2* knock-out line (referred to as *glb2* in Hebelstrup *et al.*, 2008); the *myc2-1* mutant and the 35S:MYC2 line (Dombrecht *et al.*, 2007); the 35S:JAZ1 line (Thines *et al.*, 2007); the *pJAZ1:GUS-GFP* line (Gutierrez *et al.*, 2012); the 35S*:MYC2-GUS* line (Zhai *et al*., 2013); the *pYUC4:GUS* line (Eklund *et al.*, 2011); and the *pPDF1.2:GUS* line (Koorneef *et al.*, 2008). *pgb2*-*aos1-1* double mutant lines were generated by crossing (Supplementary Fig. S1 at *JXB* online).

### Growth conditions and induction of somatic embryogenesis

Arabidopsis seeds were sterilized (70% ethanol+0.5% Triton X-100 for 15min followed by 95% ethanol for 15min) and plated on germination medium (half-strength MS; Murashige and Skoog, 1962). The plates were kept at 4 °C in the dark for 2–3 d and then transferred to a growth cabinet (20–22 °C, 16h light/8h dark photoperiod). Plants were grown until siliques were formed, ~21–28 d.

Somatic embryogenesis was promoted using a modified method based on that described by Bassuner *et al.* (2007). Immature zygotic embryos were plated on induction medium containing 2,4-dichlorophenoxyacetic acid (2,4-D) for 14 d, followed by transfer onto hormone-free development medium. Fully developed somatic embryos were counted after 9 d.

### Chemical treatments

The NO scavenger 2-(4-carboxyphenyl)-4,4,5,5-tetramethylimidazoline-1-oxyl-3-oxide (cPTIO) and the NO donor sodium nitroprusside (SNP) were applied as specified in Elhiti *et al.* (2013). Applications were performed by dispensing 10 μl of a 10 µM solution directly on the explants every other day throughout culture in the induction medium.

JA (Sigma) was dissolved in water and added to the culture medium at different concentrations as reported in the text. The JA inhibitor 1-phenyl-3-pyrazolidinone (Phenidone, Ph) was applied at a concentration of 10nM.

### Total RNA isolation and quantitative real-time PCR analysis

Total RNA was extracted with TRIzol reagent (Invitrogen), treated with DNase I (RNase-free, Promega), and utilized for cDNA synthesis with the High Capacity cDNA Reverse Transcription Kit (Applied Biosystems).

Quantitative real-time PCR was performed as described in Elhiti *et al.* (2010) using the primers listed in Supplementary Table S1. The relative level of gene expression was analyzed with the 2^−∆∆*C*t^ method described by Livak and Schmittgen (2001) using UBQ10 (AT4G05320) as a reference (Czechowski *et al.*, 2005; Hong *et al.*, 2010).

### β-Glucuronidase assays

β-Glucuronidase (GUS) histochemical staining assay was performed as described by Sieburth and Meyerowitz (1997). The somatic embryos were examined and photographed using a dissecting microscope equipped with a Leica DC500 digital camera. A minimum of 20 samples per treatment were imaged.

### IAA and JA immunolocalization

Immunolocalization of endogenous indole acetic acid (IAA) was carried out following the procedure used by Elhiti *et al.* (2013). Immunolocalization of endogenous JA was performed as described by Mielke *et al.* (2011), with some minor modifications. The plant material was fixed in 4% (w/v) 1-ethyl-3-(3–dimethylaminopropyl) carbodiimide (EDC) in phosphate-buffered saline (PBS) for 3h at room temperature. After dehydration in a graded ethanol series, the specimens were infiltrated in PEG-8 distearate containing low melting point wax (Electron Microscopy Sciences) at 45 °C. Sections (10 μm thickness) were incubated with anti-JA antibodies raised in rabbit (kindly donated by Professor House, IPK, Germany) diluted 1:1000 in PBS containing 5% (w/v) BSA and 1% (v/v) acetylated BSA (BSA_ac_; Promega). The secondary goat anti-rabbit IgG antibody conjugated with AlexaFluor594 (Invitrogen) was used according to the manufacturer’s instructions at a 1:2000 dilution. Sections were analyzed by epifluorescence microscopy.

### Statistical analysis

All experiments were performed using at least three biological replicates, and Tukey’s post-hoc test for multiple variance was used to compare differences among samples (Zar, 1999) (*P*=0.05) by the SPSS 14 statistical program.

## Results

### Repression of *PGB2* increases embryogenesis through the NO-mediated elevation in JA level

Arabidopsis somatic embryogenesis is a two-step process (Fig. 1A). Dissected zygotic embryos were cultured on a 2,4-D solid induction medium for 14 d. After 3 d, the cotyledons of the explants started swelling and embryogenic tissue became apparent at day 7. During the following days on induction medium (days 7–14), the embryogenic tissue increased in size. Embryo production was stimulated by transferring the tissue onto a hormone-free development medium. As also reported previously (Elhiti *et al.*, 2013), the term somatic embryos used in this study refers to fully developed embryos collected after 9 d on the development medium (Fig. 1A).

In wild-type (WT) tissue, inclusion of JA in the induction medium affected the number of somatic embryos in a dose-dependent fashion, with the most pronounced increase observed with 50nM JA (Fig. 1B). This concentration was used to compare the effects of JA manipulations on somatic embryos produced by the WT line and the *Pgb2* knock-out (*pgb2*) line characterized by a superior embryogenic performance (Elhiti *et al.*, 2013). In the WT, the JA stimulation of embryogenesis was reversed by Ph, a JA biosynthetic inhibitor (Farmer *et al.*, 1994; Bruinsma *et al.*, 2009), which strongly repressed embryo production when applied alone (Fig. 1C). The beneficial effect of JA on somatic embryogenesis was not observed in the *pgb2* line. To prove that the increased number of embryos observed in the *pgb2* line relative to the WT line was solely due to suppression of the gene, we overexpressed *PGB2* in the mutant line. The resulting 35S:PGB2/*pgb2* (Supplementary Fig. S2) had an embryonic yield comparable with the WT line (Fig. 1C)

The different embryonic behavior of the two lines following JA treatments was further examined in light of the following premises: PGB2 is an effective scavenger of NO (Hebelstrup *et al.*, 2008) expressed at the sites of the Arabidopsis explants producing embryogenic tissue (Elhiti *et al.*, 2013); NO accumulates preferentially in cells suppressing *PGB2*, and this accumulation is required for the enhanced embryogenic performance of the *pgb2* line (Elhiti *et al.*, 2013).

**Fig. 2.**
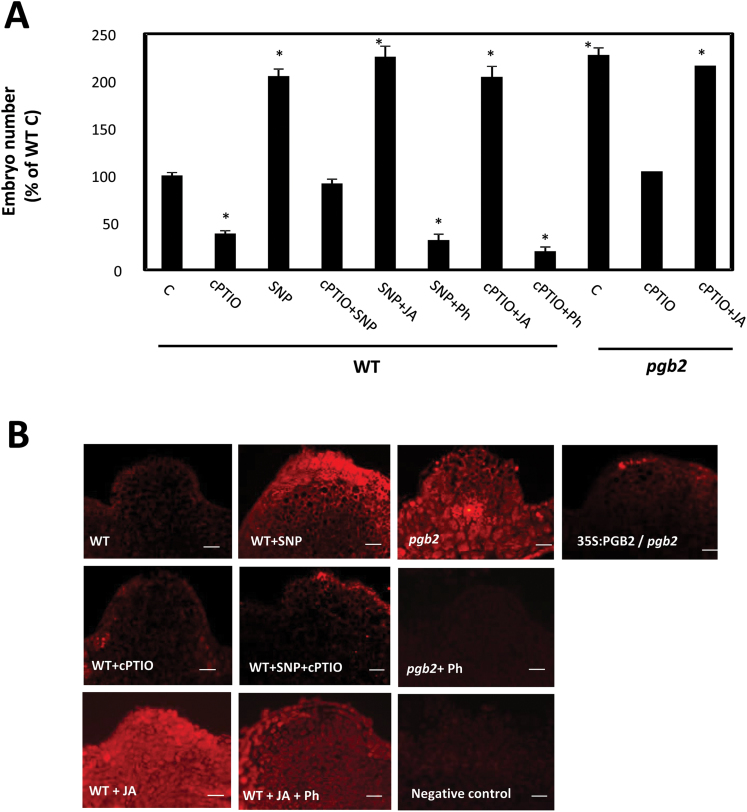
The effects of nitric oxide (NO) on somatic embryogenesis are mediated by jasmonic acid (JA). (A) Effects of applications of the NO scavenger 2-(4-carboxyphenyl)-4,4,5,5-tetramethylimidazoline-1-oxyl-3-oxide (cPTIO), the NO-releasing agent sodium nitroprusside (SNP), JA, and Phenidone (Ph) on the number of somatic embryos produced by the WT and *pgb2* lines. Values are means ±SE of at least three biological replicates. An asterisk indicates statistically significant differences (*P*≤0.005) from value of the WT (C) set at 100%. (B) Immunolocalization of JA on the embryogenic tissue arising from the cotyledons of the explants after 7 d on induction medium. Tissue was treated with SNP, cPTIO, JA, and/or Ph. Primary antibodies were omitted from the negative control. Scale bars=20 μm.

To establish an experimental baseline for our experiments, we confirmed previous results related to the effects of NO manipulations on somatic embryogenesis (Elhiti *et al.*, 2013). Applications of the NO donor SNP increased embryo production in the WT line, whereas inclusion of the NO scavenger cPTIO compromised the embryogenic process in both WT and *pgb2* lines (Fig. 2A). Compared with the WT, the number of somatic embryos more than doubled in the *pgb2* line, an observation consistent with the higher level of NO accumulating in the latter (Elhiti *et al.*, 2013). However, the beneficial effects of high NO levels (either by SNP or by suppression of *PGB2*) on somatic embryogenesis were JA dependent. In the WT line, depletion of JA by Ph in an NO-enriched environment (SNP+Ph) suppressed embryo production, whereas elevated JA levels promoted embryogenesis even in an environment depleted of NO (cPTIO+JA) (Fig. 2A). Similar results were also observed in the NO-accumulator *pgb2* line where applications of JA reversed the inhibitory effect of cPTIO (Fig. 2A). Therefore, the beneficial effects of high NO levels (by SNP or suppression of *PGB2*) on embryogenesis are mediated by JA.

We further assessed if JA accumulated preferentially in cells with increasing levels of NO. While JA quantitation cannot be performed in our system due to the reduced size of the embryos, immunolocalization of JA was conducted across the cotyledons of the explants. These are the regions generating the embryogenic tissue (Fig. 1A) and accumulating NO following suppression of *PGB2* (fig. 3A in Elhiti *et al.*, 2013). An intense JA signal was observed in NO-enriched environments (WT tissue treated with SNP and *pgb2* tissue) (Fig. 2B), while an experimental reduction in NO level by cPTIO reduced the fluorescence. Low JA signal was also observed in the 35S:PGB2/*pgb2* line. Specificity of the antibody was verified using JA and/or Ph (Fig. 2B).

Collectively these results suggest that NO induces the accumulation of JA, and heightened levels of JA favor embryogenesis in a dose–response fashion, with exogenous applications increasing embryo number only in systems with reduced levels of endogenous JA (WT line), but not in others already enriched in JA (*pgb2* line).

### NO activates the expression of genes participating in JA biosynthesis

**Fig. 3.**
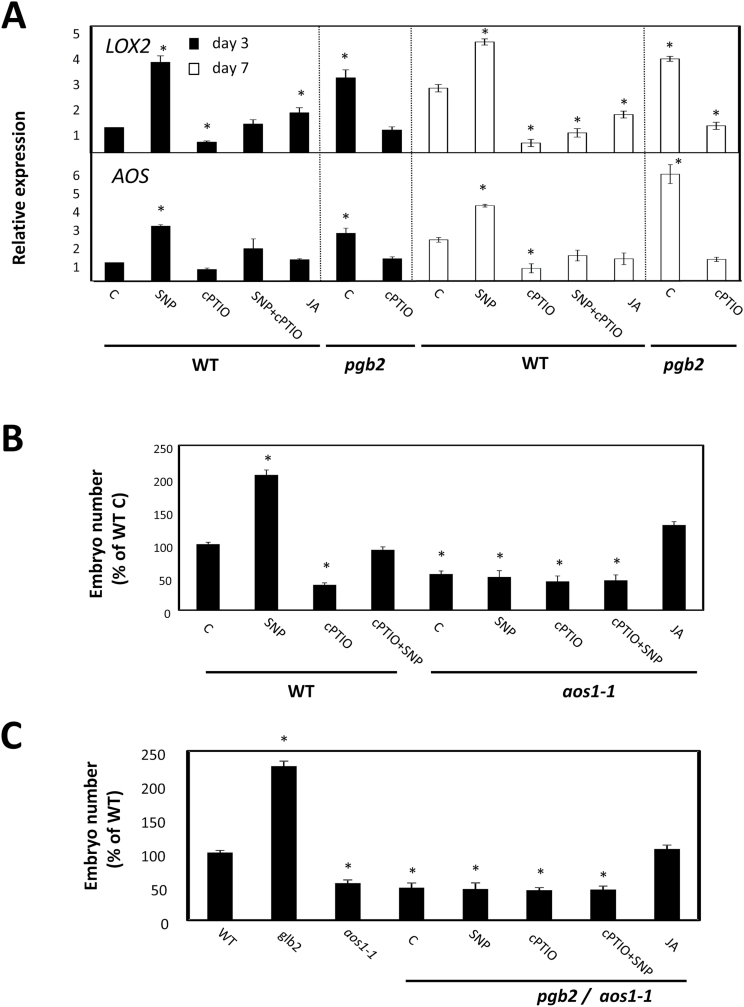
The effects of nitric oxide (NO) on jasmonic acid (JA) synthesis. (A) Expression level by quantitative (q)RT–PCR of the two JA biosynthetic genes [*LIPOXYGENASE2* (*LOX2*) and *ALLENE OXIDE SYNTHASE* (*AOS*)] at days 3 and 7 in the induction medium of somatic embryogenesis. WT and *pgb2* tissue was cultured in medium with altered levels of NO and JA using the pharmacological treatments described in Fig. 2. Values are means ±SE of at least three biological replicates and normalized to the value of the WT (C) of day 3 set at 1. Asterisks indicate statistically significant differences (*P*≤0.005) from the WT (C) value of the respective day in culture. (B) Number of somatic embryos generated from WT and *aos* plants. Explants were cultured on medium with altered levels of NO and JA. Values are means ±SE of at least three biological replicates. An asterisk indicates statistically significant differences (*P*≤0.005) from the WT (C) value set at 100%. (C) Number of somatic embryos generated from WT, *pgb2 aos*, and *pgb2/aos* double mutant plants. Explants were cultured on medium with altered levels of NO and JA. Values are means ±SE of at least three biological replicates. An asterisk indicates statistically significant differences (*P*≤0.005) from the WT value set at 100%.

*De novo* biosynthesis of JA requires the co-ordinated expression of several genes including *LYPOXYGENASE2* (*LOX2*) involved in the oxygenation of fatty acids to their hydroperoxy derivatives and *ALLENE OXIDE SYNTHASE* (*AOS*), forming unstable allene epoxides from the dehydratation of 1,3-hydroperoxy-octatrienoic acid (Wasterack and Hause, 2013). On day 3 and day 7 on induction medium (coinciding with the formation of embryogenic tissue Fig. 1A), the expression of both genes increased in NO-enriched environments (i.e. SNP-treated WT tissue and *pgb2* tissue). Consistent with these observations, a reduction of NO level by cPTIO in both WT and *pgb2* lines repressed the expression of both genes (Fig. 3A). Reintroduction of *PGB2* in the *pgb2* line reduced the expression levels of both *LOX2* and *AOS* to WT values (Supplementary Fig. S3)

The requirement for JA, and more specifically the expression of its biosynthetic gene *AOS*, as downstream components of the PGB2 and NO regulation of embryogenesis was further examined by analyzing the behavior in culture of the JA-deficient *aos1-1* mutant (Park *et al.*, 2002), and the *pgb2-aos* double mutant (Fig. 3B, C). Somatic embryo production was significantly reduced by the suppression of *AOS* independently from NO levels and *PGB2* expression. This reduction was partially reversed by exogenous JA (Fig. 3B, C).

Evidence presented here suggests that NO is a transcriptional activator of JA biosynthesis, and this activation is required for the *PGB2* regulation of somatic embryogenesis.

### Regulation of embryogenesis by JA is mediated by JAZ1 and MYC2

**Fig. 4.**
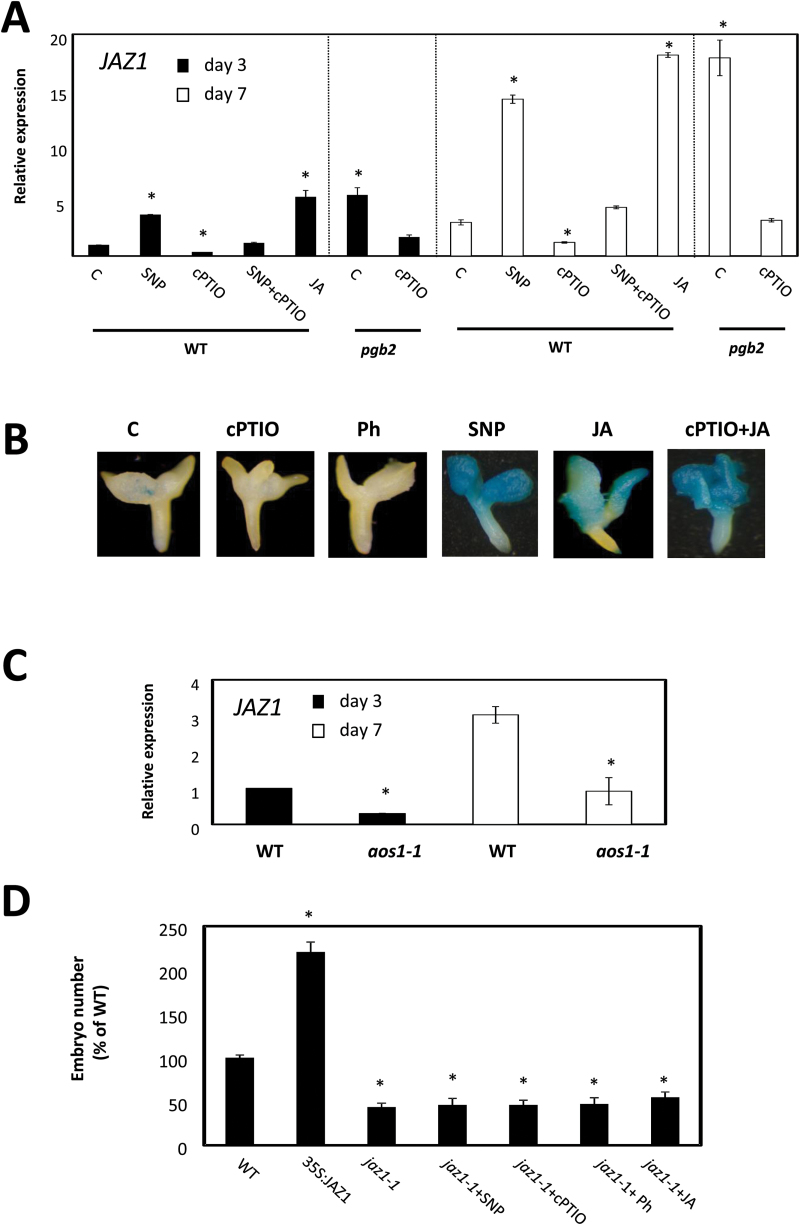
Nitric oxide (NO) and jasmonic acid (JA) affect *JAZ1*. (A) Expression level by quantitative (q)RT–PCR of *JAZ1* at days 3 and 7 in the induction medium of somatic embryogenesis. WT and *pgb2* tissue was cultured in medium with altered levels of NO and JA using the pharmacological treatments described in Fig. 2. Values are means ±SE of at least three biological replicates and are normalized to the value of the WT (C) of day 3 set at 1. An asterisk indicates statistically significant differences (*P*≤0.005) from the WT (C) value of the respective day in culture. (B) Localization patterns of *JAZ1* by GUS staining at day 7 on induction medium. Tissue was subjected to the indicated pharmacological treatments. (C) Expression level by qRT–CR of *JAZ1* in the WT and *aos* lines at days 3 and 7 on induction medium. Values are means ±SE of at least three biological replicates and are normalized to the value of the WT at day 3 set at 1. An asterisk indicates statistically significant differences (*P*≤0.005) from the WT value of the respective day in culture. (D) Number of somatic embryos generated from WT, 35S:JAZ1, and *jaz1-1* plants. Explants were cultured on media with altered levels of NO and JA. Values are means ±SE of at least three biological replicates. An asterisk indicates statistically significant differences (*P*≤0.005) from the WT value set at 100%.

JAZ1 is a JA-inducible nuclear-localized protein belonging to the larger family of the TIFY proteins (Vanholme *et al.*, 2007) integrated in several JA biotic stress responses (Niu *et al.*, 2010; Sasaki-Sekimoto *et al.*, 2013). During somatic embryogenesis, *JAZ1* expression is induced after 12h of JA application (Supplementary Fig. S4). On both day 3 and day 7 on induction medium, its expression increased in the *pgb2* line and in response to pharmacological treatments elevating NO or JA levels, while it decreased following depletion of NO (by cPTIO) (Fig. 4A). Reintroduction of *PGB2* in the *pgb2* line reduced the expression levels of JAZ1 to WT values (Supplementary Fig. S3)

The regulation of JAZ1 during the induction of embryogenesis was also confirmed using the *pJAZ1:GUS* reporter line showing a staining pattern in the apical regions of the explants, including the embryogenic tissue, mediated by NO and JA levels (Fig. 4B).

To verify further the requirement for JA for *JAZ1* expression during somatic embryogenesis, we used the JA-deficient *aos1-1* line. Compared with the WT, the expression of *JAZ1* was significantly repressed in the *aos1-1* explants on both day 3 and day 7 on induction medium (Fig. 4C).

Modulations in *JAZ1* expression have profound effects on somatic embryogenesis. While the constitutive expression of this gene in the 35S:JAZ1 line increased embryo production, its suppression in the *jaz1-1* mutant repressed embryogenesis (Fig. 4D). The fact that this repression was not rescued by manipulating the levels of NO (by SNP or cPTIO) or JA (by Ph or JA) suggests that JAZ1 is a downstream component in the regulation of embryogenesis.

Another key executor of the JA response during biotic and abiotic stress responses is MYC2, a bHLH domain-containing transcription factor (Dombrecht *et al.*, 2007). Expression of *MYC2* shows a biphasic pattern in response to JA. While initial applications of JA increase *MYC2* expression, prolonged exposure to JA repressed *MYC2* expression (Supplementary Fig. S4).

**Fig. 5.**
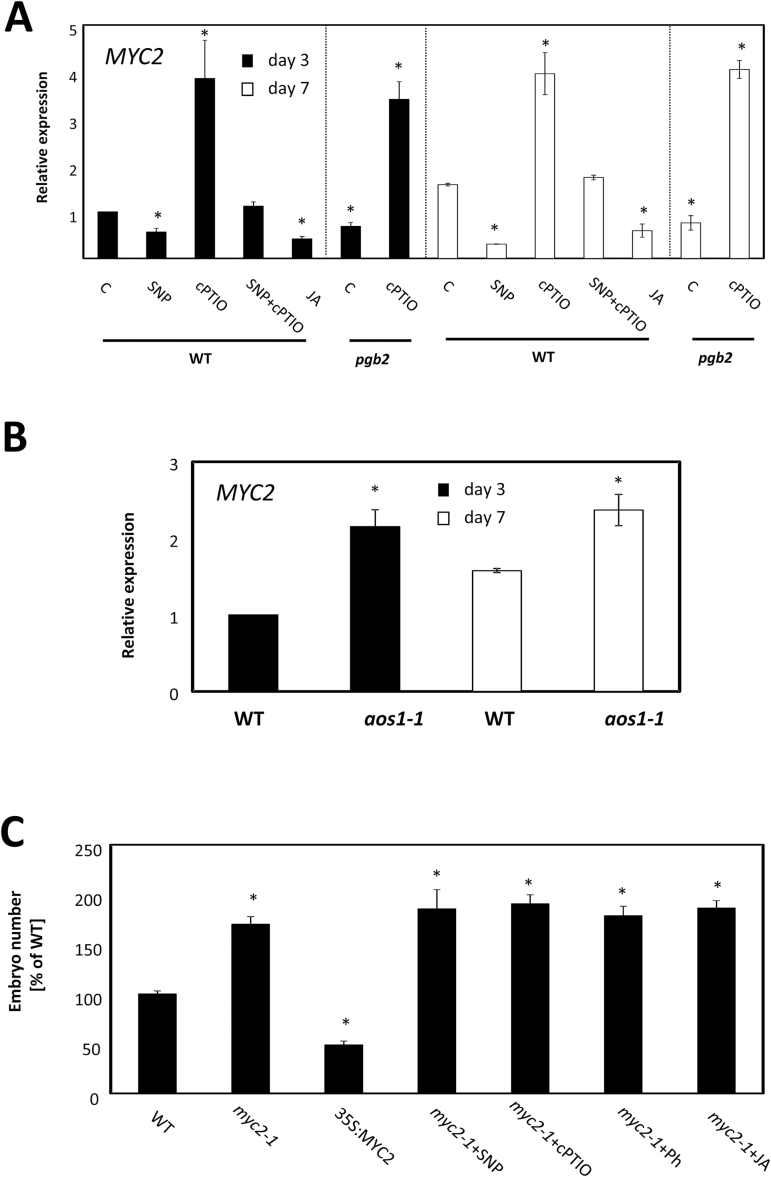
Nitric oxide (NO) and jasmonic acid (JA) affect *MYC2*. (A) Expression level by quantitative (q)RT–PCR of *MYC2* at days 3 and 7 in the medium with altered levels of NO and JA using the pharmacological treatments described in Fig. 2. Values are means ±SE of at least three biological replicates and are normalized to the value of the WT (C) of day 3 set at 1. An asterisk indicates statistically significant differences (*P*≤0.005) from the WT (C) value of the respective day in culture. (B) Expression level by qRT–PCR of *MYC2* in WT and *aos* lines at days 3 and 7 on induction medium. Values are means ±SE of at least three biological replicates and are normalized to the value of the WT at day 3 set at 1. An asterisk indicates statistically significant differences (*P*≤0.005) from the WT value of the respective day in culture. (C) Number of somatic embryos generated from WT, 35S:MYC2, and *myc2-1* plants. Explants were cultured on media with altered levels of NO and JA. Values are means ±SE of at least three biological replicates. An asterisk indicates statistically significant differences (*P*≤0.005) from the WT value set at 100%.

*MYC2* is repressed in *PGB2*-suppressed cells, and this repression promotes auxin production and favors somatic embryogenesis (Elhiti *et al.*, 2013). On both day 3 and day 7 on induction medium, *MYC2* was down-regulated in high JA and NO environments (JA- or SNP-treated tissue and *pgb2* tissue), while it was induced by cPTIO (Fig. 5A), or by the the overexpression of *PGB2* in the *pgb2* line (Supplementary Fig. S3). A significant induction of this gene also occurred in the JA-deficient *aos1-1* explants on the same days in culture (Fig. 5B). Suppression in *MYC2* in the *myc2-1* mutant increased embryogenesis in a pattern consistent with previous studies (Elhiti *et al.*, 2013). This increase, however, was not affected by pharmacological treatments altering the levels of NO or JA (Fig. 5C).

These results suggest that transcriptional regulation of *MYC2*, like *JAZ1*, plays an important role in the NO and JA control of Arabidopsis somatic embryogenesis, and that increased embryo number is favored by the up-regulation of *JAZ1* and the repression of *MYC2*.

**Fig. 6.**
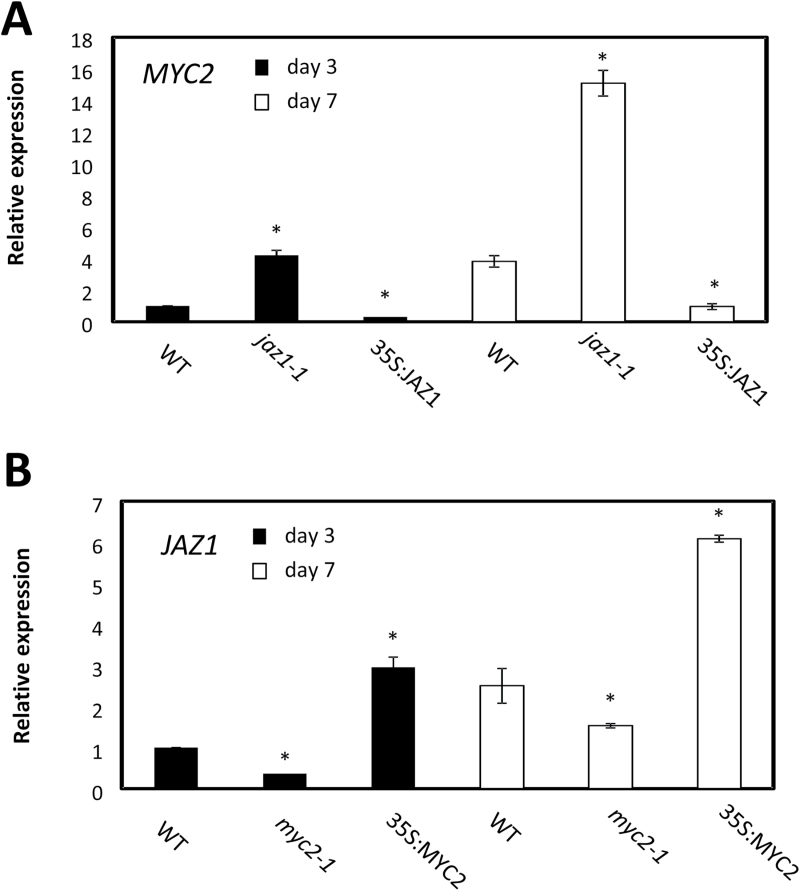
Transcriptional regulation of *MYC2* and *JAZ1*. (A) Expression level by quantitative (q)RT–PCR of *MYC2* at days 3 and 7 in the induction medium of somatic embryogenesis in the WT, *jaz1-1*, and 35S:JAZ1 lines. Values are means ±SE of at least three biological replicates and are normalized to the value of the WT of day 3 set at 1. An asterisk indicates statistically significant differences (*P*≤0.005) from the WT value of the respective day in culture. (B) Expression level by qRT–PCR of *JAZ1* at days 3 and 7 in the induction medium of somatic embryogenesis in the WT, *myc2-1*, and 35S:MYC2 lines. Values are means ±SE of at least three biological replicates and are normalized to the value of the WT of day 3 set at 1. An asterisk indicates statistically significant differences (*P*≤0.005) from the WT value of the respective day in culture.

The regulation of JAZ1 and MYC2 during the JA response in plant–pathogen interaction is complicated by feedback loops influencing the expression of both (Chini *et al.*, 2007; Thines *et al.*, 2007; Chung *et al.*, 2008; Kazan and Manners, 2013). To verify the presence of transcriptional regulatory mechanisms operating during somatic embryogenesis, the expression of the two genes was measured in lines suppressing or ectopically expressing *MYC2* and *JAZ1*. On both day 3 and day 7 on induction medium, the expression of *MYC2* was induced in the *jaz1-1* line while it was repressed in the 35S:JAZ1 line. This pattern was in contrast to that observed for *JAZ1* which was down-regulated in the *myc2-1* line and up-regulated in the 35S:MYC2 line (Fig. 6).

### JA response modulates embryogenesis by altering IAA biosynthetic genes

**Fig. 7.**
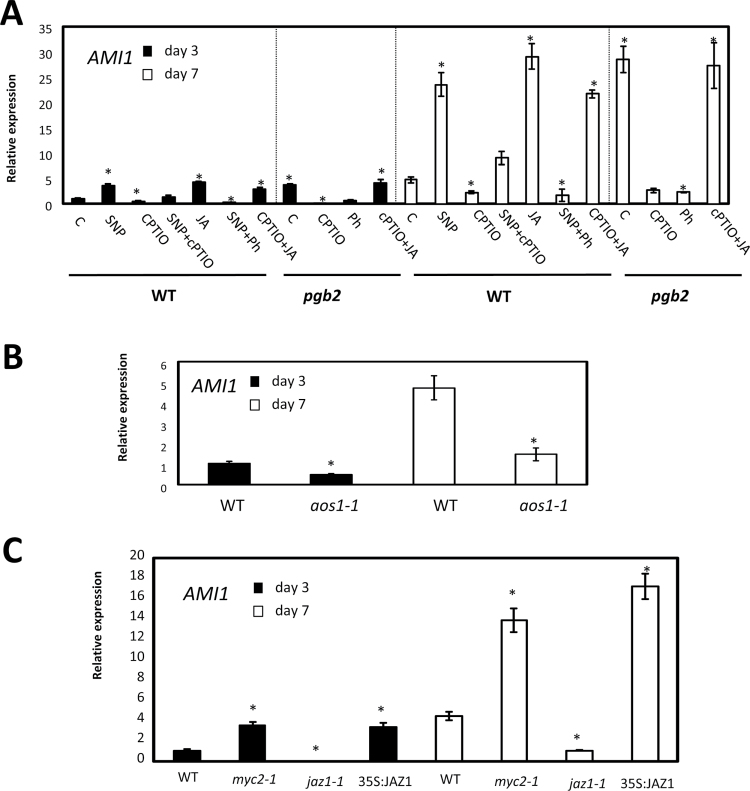
Effects of jasmonic acid (JA) and nitric oxide (NO) on *AMI1* expression. (A) Expression level by quantitative (q)RT–PCR of *AMI1* at days 3 and 7 in the induction medium of somatic embryogenesis. WT and *pgb2* tissue was cultured in medium with altered levels of NO and JA using the pharmacological treatments described in Fig. 2. Values are means ±SE of at least three biological replicates and are normalized to the value of the WT (C) of day 3 set at 1. An asterisk indicates statistically significant differences (*P*<0.005) from the WT (C) value of the respective day in culture. (B) Expression level by qRT–PCR of *AMI1* in WT and *aos* lines at days 3 and 7 on induction medium. Values are means ±SE of at least three biological replicates and are normalized to the value of the WT of day 3 set at 1. An asterisk indicates statistically significant differences (*P*<0.005) from the WT value of the respective day in culture. (C) Expression level by qRT–PCR of *AMI1* at days 3 and 7 in the induction medium of somatic embryogenesis in the WT, *myc2-1*, *jaz1-1*, and 35S:JAZ1 lines. Values are means ±SE of at least three biological replicates and are normalized to the value of the WT of day 3 set at 1. An asterisk indicates statistically significant differences (*P*<0.005) from the WT value of the respective day in culture.

During the initial phases of somatic embryogenesis, auxin is the inductive signal promoting the re-differentiation of the somatic cells and formation of embryogenic tissue (Raghavan, 2004). Previous studies showed that suppression of *PGB2* increases auxin levels in a process dependent on the concurrent suppression of *MYC2*, an inhibitor of IAA synthesis (Elhiti *et al.*, 2013). To expand this model further, we assessed whether IAA biosynthesis was regulated by the JA response during embryogenesis. Production of IAA from indole-3-acetamide is modulated by *AMIDASE1* (*AMI1*) (Pollman *et al.*, 2003). On both days 3 and 7 on induction medium, *AMI1* expression was up-regulated by all those treatments increasing NO (SNP or suppression of *PGB2*) or JA, while it was suppressed by low levels of NO (cPTIO) or JA (Ph) (Fig. 7A). In addition, a depletion of JA in an enriched NO environment (SNP+Ph) decreased the expression of this gene while an enrichment in JA level under low NO conditions (cPTIO+JA) had opposite effects (Fig. 7A). Reintroduction of *PGB2* into the *pgb2* line reduced *AMI1* expression to WT values (Supplementary Fig. S3). The JA requirement for the sustained expression of *AMI1* was also confirmed using the JA-deficient *aos1-1* line in which *AMI1* transcript levels were reduced on both day 3 and day 7 on induction medium (Fig. 7B). To assess further if *AMI1* regulation was downstream of the JA response, transcription studies were conducted in lines with altered expression of *JAZ1* and *MYC2*. The transcript levels of *AMI1* were induced by suppression of *MYC2* (*myc2-1* mutant) or ectopic expression of *JAZ1* (35S:JAZ1) (Fig. 7C). Besides *AMI1*, two other IAA biosynthetic genes: *ANTHRANILATE SYNTHASE*-α *SUBUNIT* (*ASA1*), encoding a component of the early enzyme converting chorismate to ribosyl anthralilate, and *YUCCA* (*YUC4*) converting indole-3-yl pyruvate to (indol-3-yl)acetate (Cheng *et al.*, 2006; Zhao, 2011), show a similar JA-mediated transcriptional regulation (Supplementary Figs S3, S5, S6). The expression of both genes occurs mainly in the cotyledonary regions of the explant originating the embryogenic tissue (Supplementary Figs S5, S6).

**Fig. 8.**
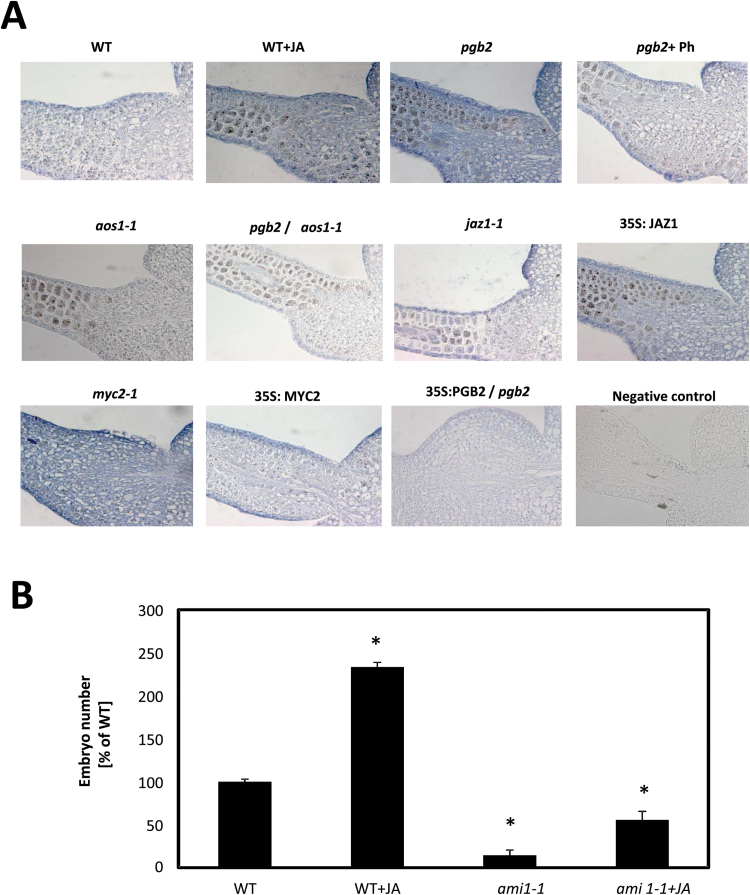
Auxin and somatic embryogenesis. (A) Immunolocalization of IAA along the cotyledons of explants collected at day 7 on induction medium. Treatments described in Figs 6 and 7 were utilized. Primary antibody was omitted from the negative control section. (B) Number of somatic embryos generated from the WT and the *ami1-1* mutant treated with JA. Values are means ±SE of at least three biological replicates. An asterisk indicates statistically significant differences (*P*≤0.005) from the WT value set at 100%.

Immunolocalization of the auxin IAA in the embryogenic tissue arising from the cotyledons of the zygotic explants confirmed the localization pattern of the biosynthetic genes (Fig. 8A). Tissues with elevated JA levels (WT+JA or *pgb2*) or subjected to conditions enhancing the JA response (*myc2-1* mutant or 35S:JAZ1) had strong IAA signals. A reduction in IAA level occurred either in tissue depleted in JA level (*pgb2* +Ph, *aos1-1, pgb2/aos1-1*), following conditions repressing the JA response (*jaz1-1* and 35S:MYC2), or by the reintroduction of *PGB2* in the *pgb2* line (Fig. 8A). The requirement for IAA production in the JA regulation of somatic embryogenesis was further verified using the *ami1-1* mutant, which significantly limits the beneficial effect of JA on embryo number (Fig. 8B).

**Fig. 9.**
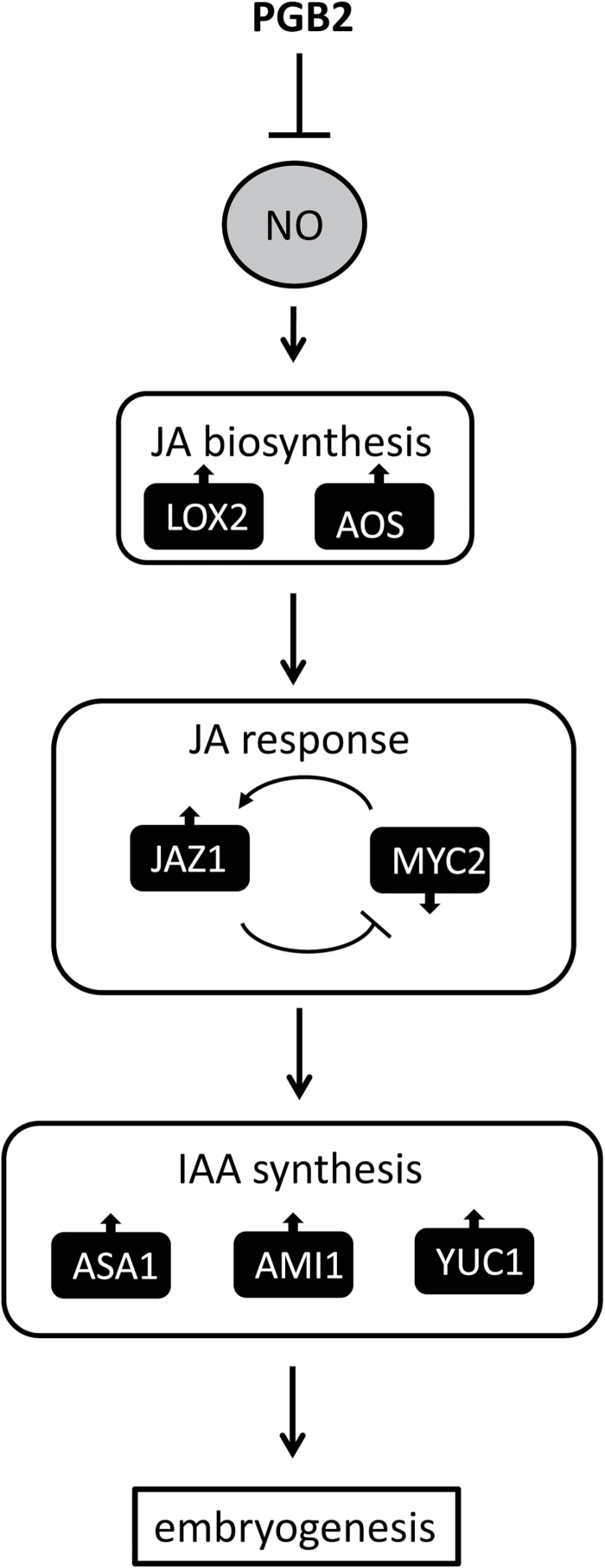
Suggested model describing the interaction among PGB2, NO, JA, and IAA during the induction phase of somatic embryogenesis in Arabidopsis.

Collectively, these data suggest that increased NO levels, resulting either from pharmacological treatments or by suppression of *PGB2*, increase JA production through the transcription of two key biosynthetic enzymes, *LOX2* and *AOS*, which activate the JA response (Fig. 9). Key events in this response are the induction of *JAZ1* and the repression of *MYC2* which promote the accumulation of IAA within the embryogenic tissue and the formation of somatic embryos.

## Discussion

Generation of embryos *in vitro* is rendered possible by the inherent ability of plant cells to embark on novel developmental programs when subjected to specific changes in environmental conditions. This concept is best exemplified in Arabidopsis, where isolated zygotic embryos cultured on induction medium produce embryogenic tissue from the cotyledons; an event which becomes apparent after 3–7 d. The process is stimulated by the auxin 2,4-D, the inductive signal responsible for the de-differentiation of the cotyledon cells and proliferation of embryogenic cells (Raghavan, 2004). The embryogenic tissue is composed of immature embryos which are allowed to grow further on a development medium devoid of auxin (Fig. 1A). Production of somatic embryos in Arabidopsis is facilitated by the suppression of the phytoglobin PGB2, an effective NO scavenger (Hebelstrup *et al.*, 2008). Explants suppressing *PGB2* accumulate NO within the embryogenic tissue and this promotes the formation of somatic embryos (Elhiti *et al.*, 2013). This enhanced embryogenic ability was solely due to the suppression of *PGB2*, as the reintroduction of *PGB2* in the *pgb2* line decreased the number of embryos to WT values (Fig. 1C)

In plants, NO participates in diverse physiological functions and operates at the interphase of many hormone-mediated responses (Neill *et al.*, 2003; Wilson *et al.*, 2008; Hancock *et al.*, 2011; Freschi, 2013; Simontacchi *et al.*, 2013). Involved in many developmental responses ranging from leaf expansion, root growth, and senescence (Wendehenne *et al.*, 2001; Yadav *et al.*, 2013), NO is tightly linked to JA in wounding and plant pathogen interaction (Grun *et al.*, 2006; Wang *et al.*, 2013). Having emerged as a downstream modulator in the JA response, NO has also been shown to contribute to JA synthesis (Mur *et al.*, 2012). A rapid induction in JA level following inoculation with *B. cinerea* was observed in Arabidopsis plants accumulating NO (Hebelstrup *et al.*, 2012). Interactions between NO and JA signaling have only been investigated during post-embryonic growth, with no information related to embryogenesis. Here we show that NO influences the number of somatic embryo produced by modulating the JA level and response, and that these effects can be integrated in the *PGB2* regulation of somatic embryogenesis (Elhiti *et al.*, 2013).

Reports on exogenous JA applications during *in vitro* embryogenesis are scarce and contradictory. While promoting the formation of protocorm bodies, the equivalent of somatic embryos in orchids (Teixeira da Silva, 2012), applications of JA repress callus growth and somatic embryogenesis in *Medicago sativa* (Kepezynska and Zielinska, 2006). These discrepancies might be ascribed, at least in part, to differences in endogenous JA content, as also observed in our system. Applications of JA (50nM) enhanced embryo production in the WT line, characterized by a low JA signal in the embryogenic tissue, but not in the *pgb2* line accumulating high levels of JA and producing a higher number of embryos (Figs 1C, 2). In this latter line, a depletion in JA level by Ph, an effective lipoxygenase inhibitor of JA synthesis (Farmer *et al.*, 1994; Bruinsma *et al.*, 2009), reduces embryo number, and this reduction is not due to the toxic effect of the inhibitor since embryo production is re-established by JA+Ph (Fig. 1C). Therefore, the enhanced embryogenic performance of the *pgb2* line is most probably the result of a higher JA content within the embryogenic tissue.

Jasmonic acid mediates the NO and PGB2 regulation of somatic embryogenesis. First, the promotive effect of high NO environments (by SNP or suppression of *Pgb2*) on embryo formation is abolished by depleting JA with Ph. Consistent with this result, the reduction in embryo number under conditions of low NO (by cPTIO) is fully reversed by applications of JA (Fig. 2A). Secondly, manipulations of the NO content influence JA levels in the embryogenic tissue, with SNP increasing the intensity of the signal and cPTIO reducing it (Fig. 2B). Thirdly, NO induces the expression of two important JA biosynthetic genes, *LOX2* and *AOS*, at both day 3 and day 7 on the induction medium (Fig. 3A), corresponding to the appearance of the embryogenic tissue (arrows in Fig. 1A). Among the 13 *LOX* genes found in Arabidopsis, LOX2 is the major contributor (~75%) to the total JA produced after wounding (Bell *et al.*, 1995; Schommer *et al.*, 2008). A similar result was also obtained for AOS, which catalyzes the dehydration of the hydroperoxide to an unstable allene oxide in the JA biosynthetic pathway. Suppression of this gene almost completely abolishes the accumulation of JA following wounding (Park *et al.*, 2002; Von Malek *et al.*, 2002).

Unequivocal evidence for the involvement of JA as a downstream component of the NO mediation of somatic embryogenesis is apparent from the behavior of the JA-deficient *aos1-1* line. Suppression of *AOS* reduces embryo production and this effect cannot be reversed by treatments altering NO levels, including SNP which under normal circumstances increases embryo production (Fig. 3B). The identical response observed in the *pgb2*/*aos1-1* double mutant line, showing a reduction in embryogenesis independent of NO levels (Fig. 3C), confirms that JA is an integral mediator of NO generated by the suppression of *PGB2*.

The majority of JA responses observed during pollen development, wounding, and biotic stresses rely on a complicated interaction of downstream elements including JAZ1 and MYC2 (Kazan and Manners, 2013). In an uninduced state, the JA response is subdued by several repressors which are rapidly polyubiquinated and degraded via the 26S proteasome degradation pathway following JA treatments (Chini *et al.*, 2007). A rise in JA induces MYC2, a bHLH domain-containing transcription factor (Dombrecht *et al.*, 2007), which initiates a transcriptional cascade characterizing the ‘early JA response’. Within this cascade, genes participating in insect defense/wounding responses are up-regulated (Guerineau *et al.*, 2003), while others regulating auxin biosynthesis and responses to pathogens are repressed (Turner *et al.*, 2002; Wasternack and Hause, 2013) (diagram in Supplementary Fig. S7). The induction of MYC2 is transient, lasting only a few hours, as MYC2 also up-regulates its own repressor, *JAZ1* (Chini *et al.*, 2007). Suppression of *MYC2* by JAZ1 triggers the ‘late JA response’ characterized by the repression of the genes *VEGETATIVE STORAGE PROTEINS 1* and *2* (*VSP1* and *VSP2*), induction of the *PLANT DEFENSIN 1* (*PDF1*) gene through the repression of the AP2/ERF transcription factor *ORA59* and *ETHYLENE RESPONSIVE FACTOR1* (*ERF1*), and the restoration of auxin synthesis through the de-repression of *PLETHORA 1* and *2* (*PLT1* and *PLT2*; Supplementary Fig. S8) (Kazan and Manners, 2013). Therefore, the temporal separation of ‘early’ and ‘late’ JA responses is controlled by the MYC2–JAZ1 feedback regulatory mechanisms (diagram in Supplementary Fig. S7).

Several pieces of evidence presented here suggests that an ‘early’ and a ‘late’ JA response occurs during somatic embryogenesis (as denoted by the biphasic profile of *MYC2* expression, and the late increase in JAZ1 expression following JA treatments; Supplementary Fig. S4). Furthermore, the increased number of embryos produced by the extended applications of JA in the induction medium is possibly the consequence of the ‘late’ response characterized by the repression of *MYC2* (Fig. 5; Supplementary Fig. S4), the increase in *JAZ1* expression (Fig. 4; Supplementary Fig. S4), and the characteristic expression profile of genes involved in insect defense, pathogen response, and auxin synthesis (Supplementary Fig. S7). Within the ‘late’ response, suppression of MYC2 and up-regulation of JAZ1, possibly through a feedback mechanism (with MYC2 inducing *JAZ1* and JAZ1 repressing *MYC2*) (Fig. 6), are essential for enhanced embryogenic output. Suppression of MYC2 directly via JA or indirectly via NO (by SNP treatments or suppression of *PGB2*) (Fig. 5A) increases the number of Arabidopsis somatic embryos (Fig. 5C). Consistent with this behavior, treatments reducing JA levels (by cPTIO or by suppression of *AOS*) up-regulate *MYC2* (Fig. 5B) and compromise embryogenesis. The inefficacy of NO and JA manipulations to reduce embryo yield in a *my*c2-1 background confirms MYC2 as a downstream component of the NO and JA response (Fig. 5C).

Unlike *MYC2*, a direct or indirect rise in JA (by SNP or suppression of *PGB2*) induces *JAZ1* especially within the cotyledons of the explants producing embryogenic cells (Fig. 4B) and favors the production of somatic embryos (Fig. 4D). The expression of *JAZ1* is repressed in environments depleted in JA (by cPTIO or by suppression of *AOS*), a condition inhibiting embryogenesis (Fig. 4A, C). As also observed for MYC2, NO and JA treatments have no effects on the embryogenic performance of the *jaz1-1* line (Fig. 4D), thus placing JAZ1 as downstream of NO and JA.

The beneficial effects of *MYC2* suppression and *JAZ1* induction on somatic embryogenesis are linked to a rise in the level of auxin, the inductive signal triggering the de-differentiation of the cotyledon cells and the production of embryogenic tissue (Raghavan, 2004). Two key transcription factors linked to auxin production, PLETHORA 1 and 2 (Pinon *et al.*, 2013), as well as several auxin biosynthetic genes including *ASAI*, converting chorismate to ribosyl anthranilate during the early steps of IAA synthesis, *YUC4*, converting indole-3-oyl pyruvate to (indol-3-oyl)acetate, and *AMI1*, producing IAA from indole-3-acetamide (Pollman *et al.* 2003), are induced in NO- and JA-enriched environments and repressed by conditions depleting NO and JA (Fig. 7; Supplementary Figs S5, S6, S8). The observation that their expression is increased by JA (regardless of the levels of NO, suppression of *MYC2*, or induction of *JAZ1*) places these genes downstream of the NO response mediated by JA. While the involvement of MYC2 in the regulation of these genes during embryogenesis confirms previous studies (Elhiti *et al.*, 2013), that of JA and JAZ1 is novel and opens up new avenues for improving propagation methods in recalcitrant species. The embryogenic tissue-specific localization pattern of both *ASA1* and *YUC4* (Supplementary Figs S5, S6) suggests that regulation of these genes might be specific to the acquisition of embryogenic competence of the cotyledon cells, and might be related to the high levels of IAA observed in these areas (Fig. 8).

Collectively, these data suggest that mechanisms modulating late JA responses post-embryonically are integrated in the PGB2 and NO regulation of *in vitro* embryogenesis. In the proposed model (Fig. 9), a rise in NO due to suppression of *PGB2* (or pharmacological treatments) induces the expression of the JA biosynthetic genes *LOX2* and *AOS* and elevates JA along the cotyledons of the explants generating embryogenic tissue. JA evokes a response suppressing *MYC2* and inducing *JAZ1*. This response, possibly reinforced by transcriptional feedback mechanisms between the two genes, up-regulates several auxin biosynthetic genes, leading to an increase in auxin levels. Auxin is required for the formation and proliferation of the embryogenic tissue, and ultimately for the successful production of the somatic embryos.

## Supplementary data

Supplementary data are available at *JXB* online.

Figure S1. Characterization of the *pgb2*/*aos1-1* double mutant lines.

Figure S2. Characterization of the 35S:*PGB2*/*pgb2* line

Figure S3. Expression of jamsonic acid- and auxin-related genes in the 35S:PGB2/*pgb2* line

Figure S4. Effects of JA applications on *myc2*-1 and *jaz1-1*.

Figure S5. Jasmonic acid (JA) and nitric oxide (NO) affect *ASA1* expression and localization.

Figure S6. Jasmonic acid (JA) and nitric oxide (NO) affect *YUC4* expression and localization.

Figure S7. Diagram showing the early and late JA responses.

Figure S8. Expression levels of *PLETHORA 1* and *2* in day 7 explants.

Table S1. Primer list.

Supplementary Data
