# Supplementary material for: Jasmonic acid is a downstream component in the modulation of somatic embryogenesis by Arabidopsis Class 2 phytoglobin
Source: J Exp Bot. 2016 Mar 9;67(8):2231–46. doi: 10.1093/jxb/erw022 (PMC4809281; doi:10.1093/jxb/erw022)
Supplement: Supplementary Data [file supp_67_8_2231__index.html]

Jasmonic acid is a downstream component in the modulation of somatic embryogenesis by Arabidopsis Class 2 phytoglobin — Jasmonic acid is a downstream component in the modulation of somatic embryogenesis by Arabidopsis Class 2 phytoglobin — Supplementary Data 

# Jasmonic acid is a downstream component in the modulation of somatic embryogenesis by Arabidopsis Class 2 phytoglobin

## Supplementary Data

Data files

- supplementary\_figures\_S1\_S8\_Table\_S1.pdf - Supplementary Data
