# Supplementary material for: Jasmonic acid is a downstream component in the modulation of somatic embryogenesis by Arabidopsis Class 2 phytoglobin
Source: J Exp Bot. 2016 Mar 9;67(8):2231–46. doi: 10.1093/jxb/erw022 (PMC4809281; doi:10.1093/jxb/erw022)
Supplement: Supplementary Data [file supp_erw022_supplementary_figures_S1_S8_Table_S1.pdf]

Jasmonic acid is a downstream component in the modulation of somatic embryogenesis by the Arabidopsis class 2 phytoglobin

Mohamed M. Mira, Owen SD Wally, Mohamed Elhiti, El-Shanshory Adel, Dhadi S. Reddy, Robert D. Hill and Claudio Stasolla

Supplemental Figures and Tables

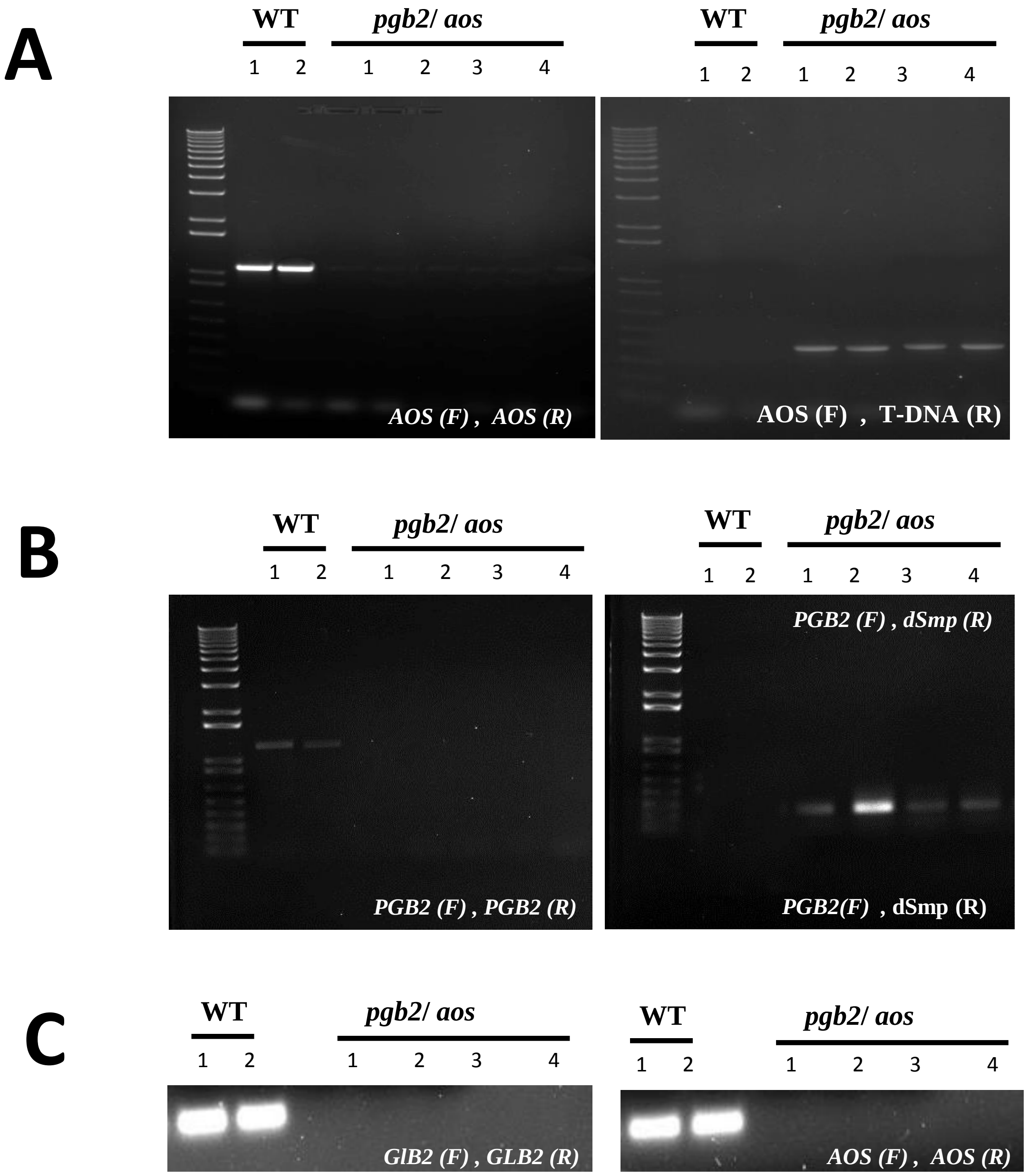

Supplemental Fig.1 . Characterization of *pgb2/ aos* double mutant lines produced by crossing the *pgb2* T-DNA line and the *aos* T-DNA line (SALK 017756). **(A)** PCR analysis of DNA to detect *AOS* (left panel) and the T-DNA insertion disrupting *AOS* (right panel). **(B)** PCR analysis of DNA to detect *PGB2* (left panel) and the T-DNA insertion disrupting *Pgb2* (dSmp) (right panel). **(C)** PCR analysis of cDNA synthesized from RNA of tissue cultured for 7 days on induction medium. Primers are shown at the bottom right corner of each gel. Primers used for amplification: AOS(F), 5'-CGAGAAATTAACGGAGCTTCC-3'; AOS(R) , 5'-CTAACCGGAGGCTACCGTATC-3'; TDNA(R), 5'-ATT TTG CCG ATT TCG GAAC-3'; PGB2(F), 5'-CTCAAAGCTCATGCTGTTAAAGTC-3'; PGB2(R), TGTGTCAGCCACTACCACCT-3';
dSmp(R), 5'-ACCGTCGACTACCTTTTTTCTTGTAGTG-3.

Supplemental Fig. 1

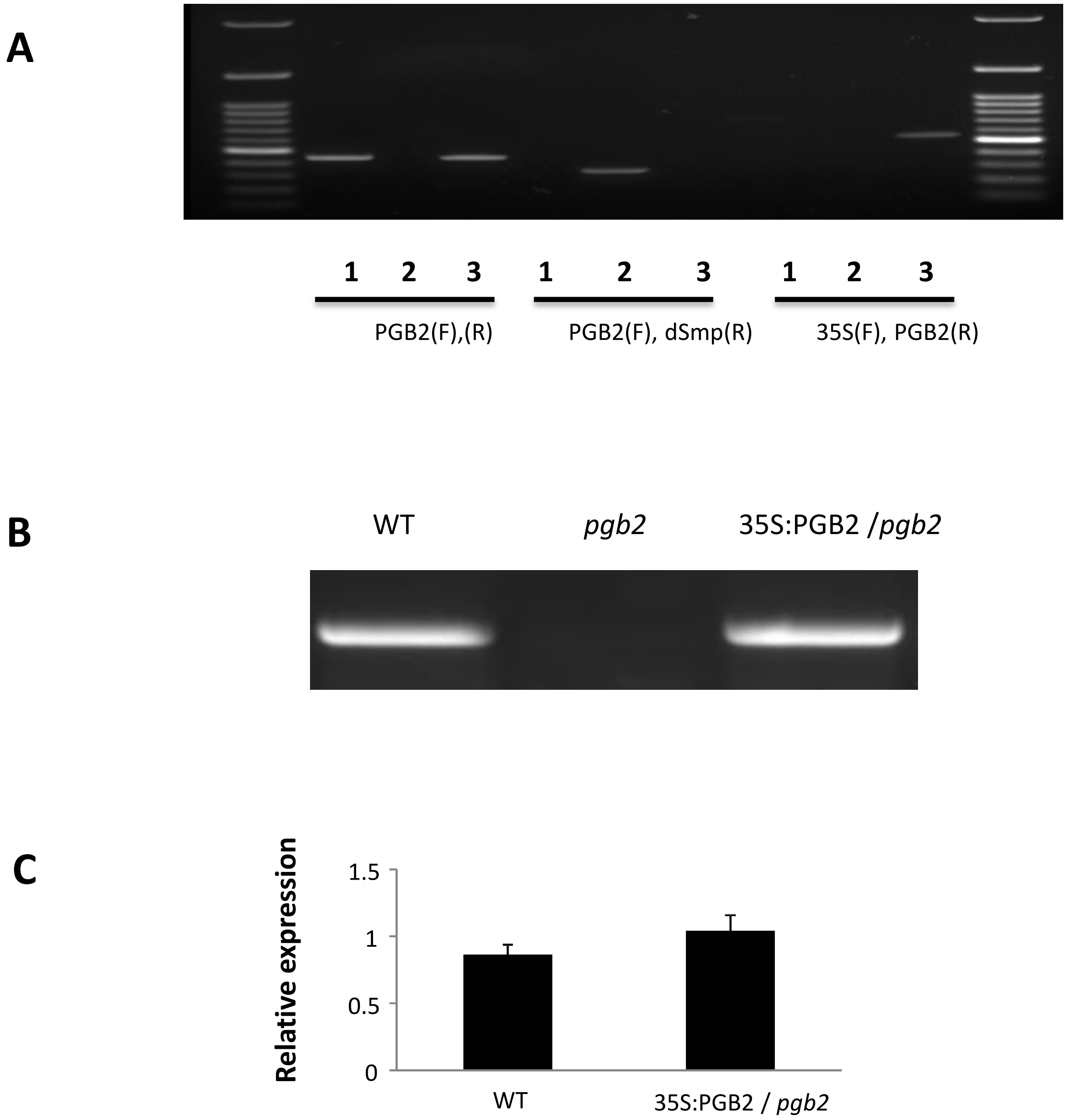

Supplemental Fig.2 . Characterization of the 35S:PGB2 / *pgb2* line produced by introducing *PGB2,* driven by the 35S promoter, into the *pgb2* line. **(A)** PCR analysis of DNA to detect the presence of PGB2 [PGB2 (F), (R) primers]; the T-DNA insertion disrupting *PGB2* (dSmp) [PGB2(F), dSmp(R) primers], and the 35S:PGB2 insert [35S (F), PGB2(R) primers]. 1, WT; 2 *pgb2;* 3, 35S:PGB2 / *pgb2.* Primers used for amplification : PGB2(F), 5'-ATGGGAGAGATTGGGTTTACAG -3'; PGB2(R), 5'-TTATGACTCTTCTTGTTTCATCTCG-3'; 35S(F), 5'-CTATCCTTCGCAAGACCCTTC-3'; and dSmp(R), 5'-ACCGTCGACTACCTTTTTTCTTGTAGTG-3. **(B)** Amplification of PGB2 by PCR from cDNA prepared from samples at 7 days on induction medium. Primers used: 5'-CTCAAAGCTCATGCTGTTAAAGT-3' and 5'- TGTGTCAGCCACTACCACCT-3'. **(C)** Relative expression of PGB2 in WT and 35S:PGB2 \ *pgb2*tissues from samples at 7 days on induction medium. Primers used: 5'- CTCAAAGCTCATGCTGTTAAAGT-3' and 5'- TGTGTCAGCCACTACCACCT-3'.

Supplemental Fig. 2

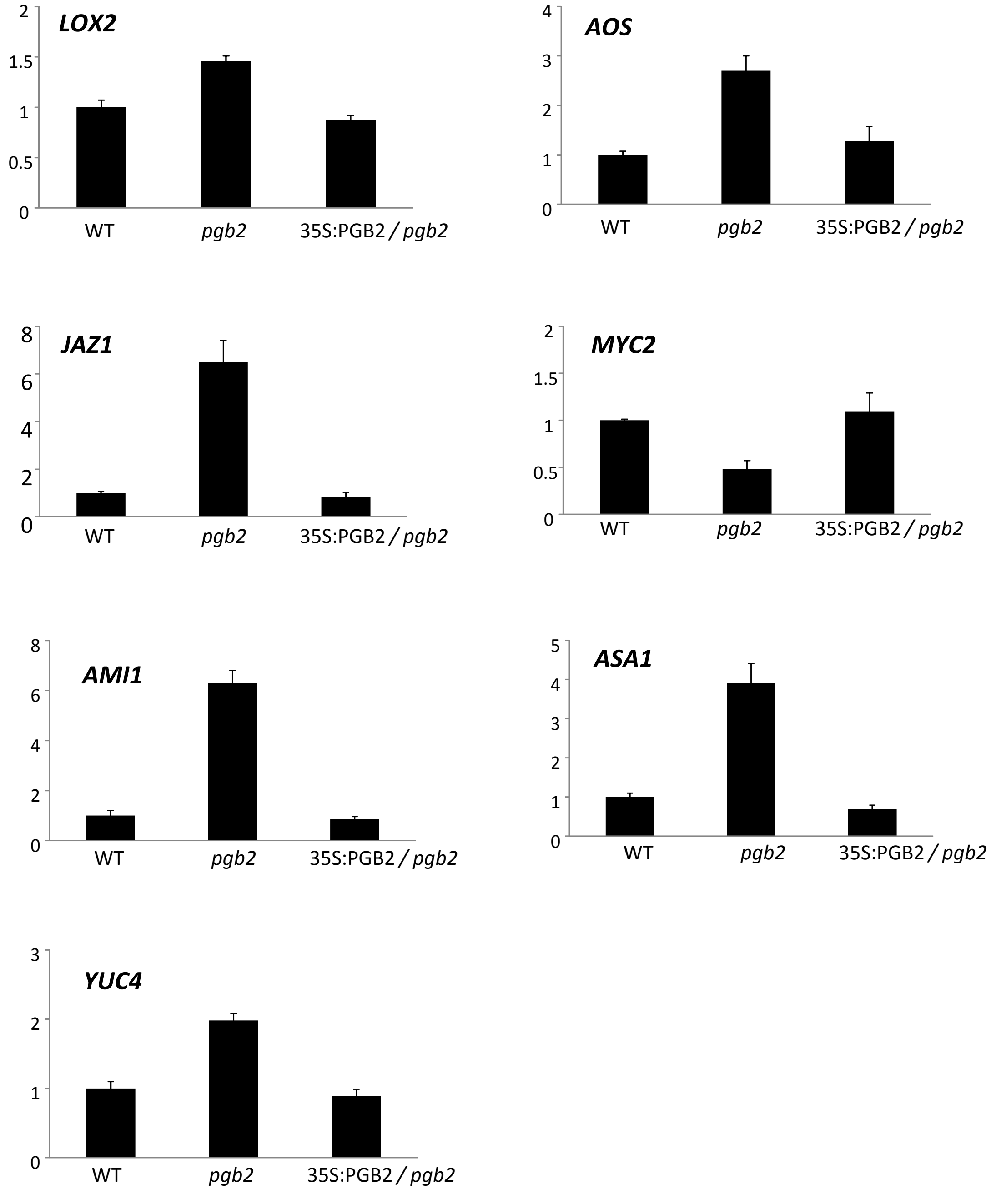

Supplemental Fig. 3. Expression level by quantitative (q)RT-PCR of several genes participating in jasmonic acid and auxin synthesis and signaling (see text for the names of the genes) in the WT, *pgb2,* and 35S:*PGB2* / *pgb2* lines at day 7 on induction medium. Values are means ± SE of at least three biological replicates and are normalized to the value of WT set at 1.

Supplemental Fig. 3

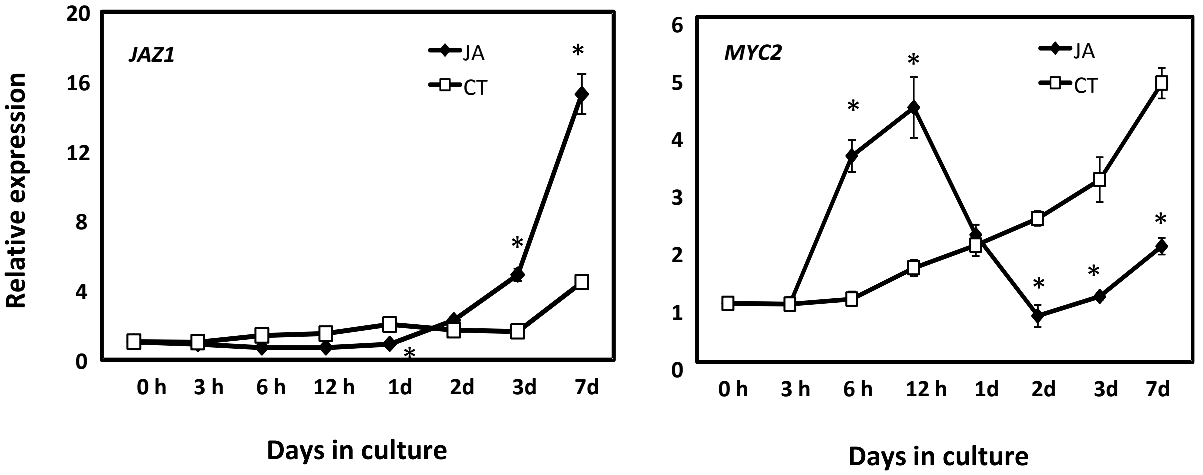

Supplemental Fig. 4. Effects of JA on the expression level of *JAZ1* and *MYC2* . Explants were cultured for 7 days on induction medium and then exposed to JA for the following 7 days. Values are means + SE of at least three biological replicates and are normalized to the value of control at time 0 set at 1. * indicates statistically significant differences (P≤ 0.005) from the control value of the respective time in culture.

Supplemental Fig. 4

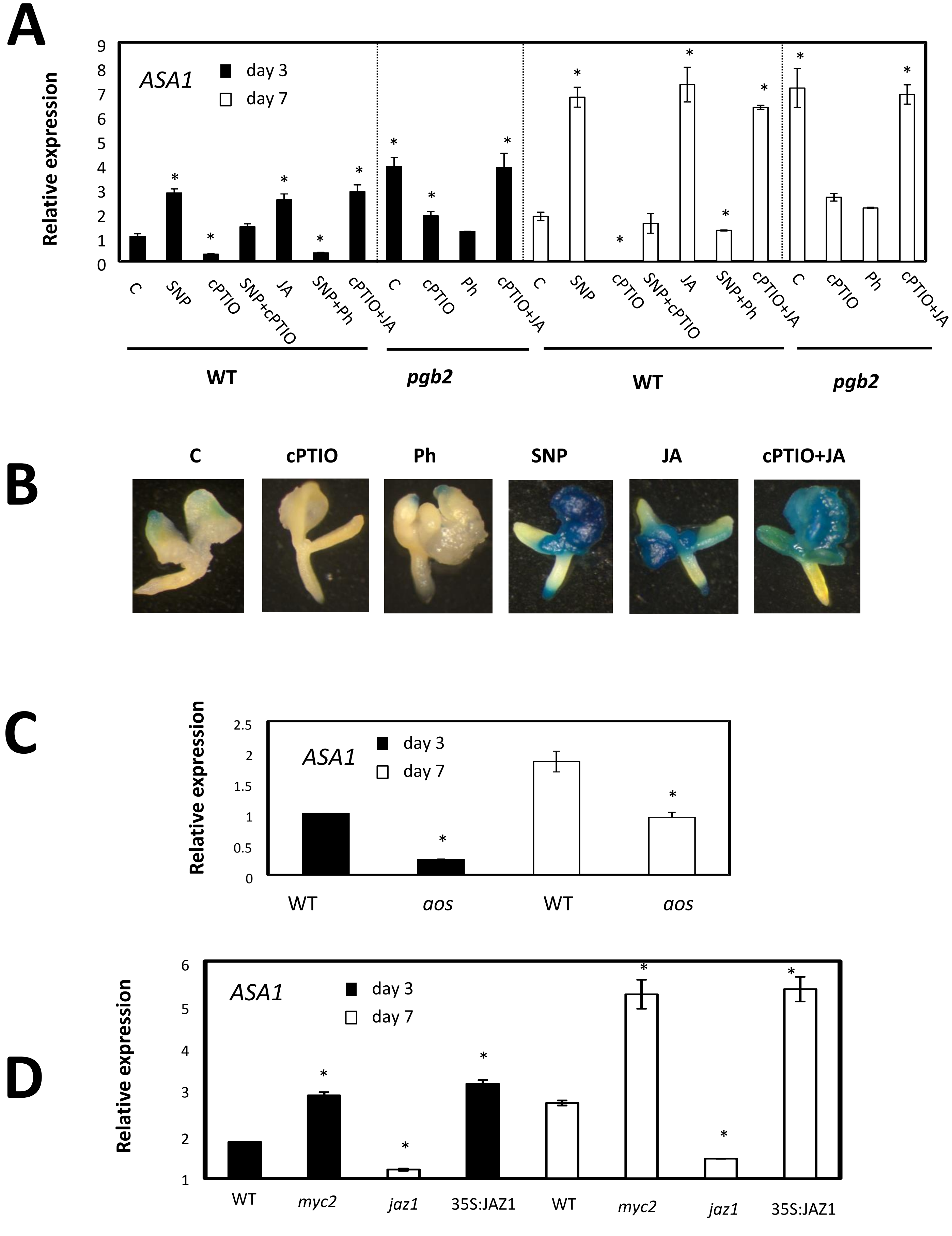

Supplemental Fig. 5

Supplemental Figure 5. Effects of jasmonic acid (JA) and nitric oxide (NO) on *ASA1* expression and localization. **(A)** Expression level by quantitative (q)RT-PCR of the auxin biosynthetitic gene *anthranilate synthase-a subunit* (*ASA1*) at day 3 and 7 in the induction medium of somatic embryogenesis. WT and *pgb2* tissues were cultured in media with altered levels of NO and JA using pharmacological treatments described in Fig. 2. Values are means ± SE of at least three biological replicates and are normalized to the value of WT(C) of day 3 set at 1. * indicates statistically significant differences (P≤ 0.005) from the WT(C) value of the respective day in culture. **(B)** Localization patterns of *ASA1* by GUS staining at day 7 on induction medium. Tissue was subjected to the indicated pharmacological treatments. **(C)** Expression level by quantitative (q)RT-PCR of *ASA1* in WT and *aos* lines at day 3 and 7 on induction medium. Values are means ± SE of at least three biological replicates and are normalized to the value of WT of day 3 set at 1. * indicates statistically significant differences (P≤ 0.005) from the WT value of the respective day in culture. **(D)** Expression level by quantitative (q)RT-PCR of *ASA1* at day 3 and 7 in the induction medium of somatic embryogenesis in the WT, *myc2*, *jaz1*, and 35S:JAZ1 lines. Values are means ± SE of at least three biological replicates and are normalized to the value of WT of day 3 set at 1. * indicates statistically significant differences (P≤ 0.005) from the WT value of the respective day in culture.

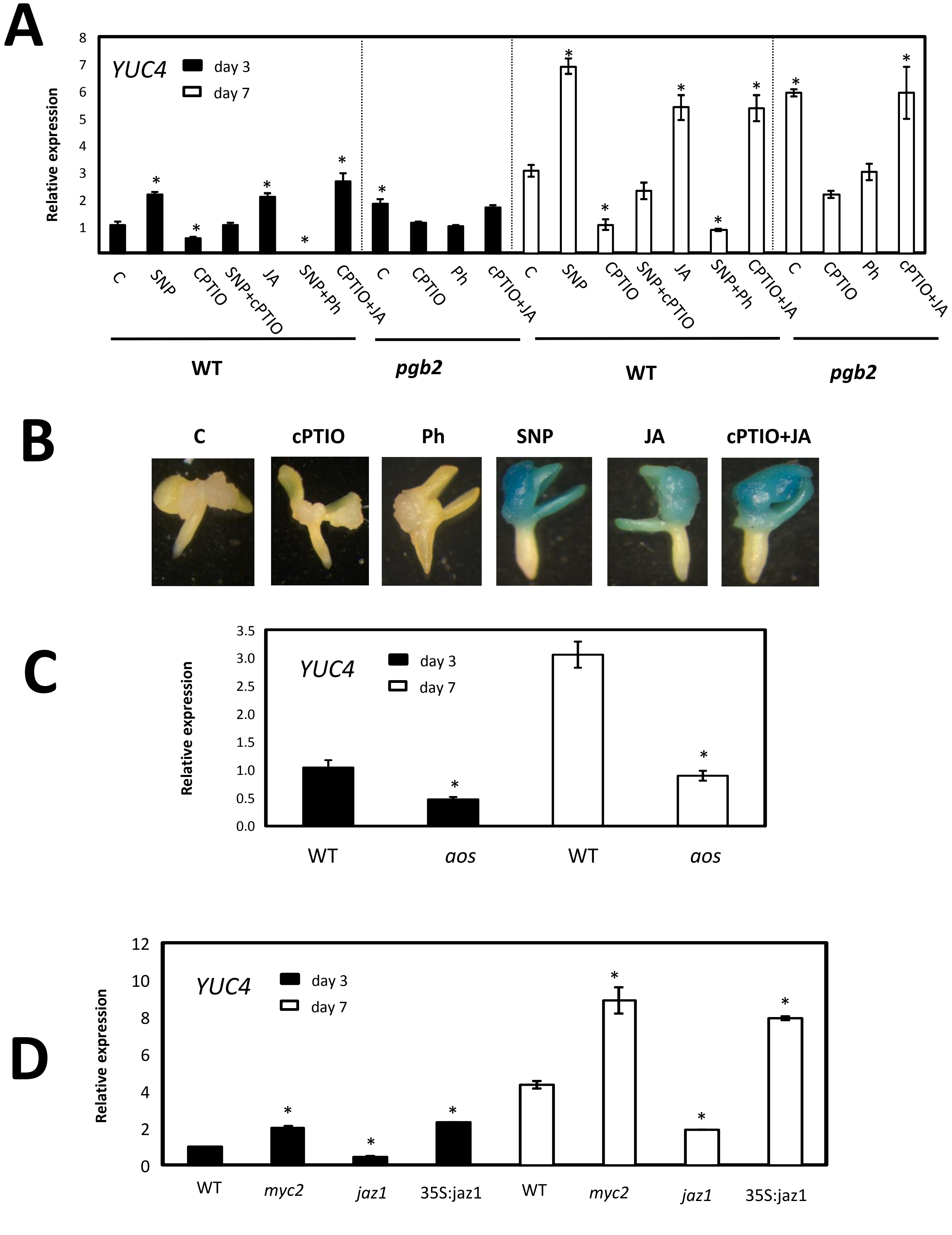

Supplemental Fig. 6

Supplemental Fig. 6. Effects of jasmonic acid (JA) and nitric oxide (NO) on *YUC4* expression and localization. **(A)** Expression level by quantitative (q)RT-PCR of *YUC4* at day 3 and 7 in the induction medium of somatic embryogenesis. WT and *pgb2* tissues were cultured in media with altered levels of NO and JA using pharmacological treatments described in Fig. 2. Values are means $\pm$ SE of at least three biological replicates and are normalized to the value of WT(C) of day 3 set at 1. * indicates statistically significant differences ($P \leq 0.005$) from the WT(C) value of the respective day in culture.
(B) Localization patterns of *YUC4* by GUS staining at day 7 on induction medium. Tissue was subjected to the indicated pharmacological treatments.

(C) Expression level by quantitative (q)RT-PCR of *YUC4* in the WT and *aos* line at day 3 and 7 on induction medium. Values are means $\pm$ SE of at least three biological replicates and are normalized to the value of WT of day 3 set at 1. * indicates statistically significant differences ($P \leq 0.005$) from the WT value of the respective day in culture. **(D)** Expression level by quantitative (q)RT-PCR of *YUC4* at day 3 and 7 in the induction medium of somatic embryogenesis in the WT, *myc2*, *jaz1*, and 35S:JAZ1 lines.
Values are means $\pm$ SE of at least three biological replicates and are normalized to the value of WT of day 3 set at 1. * indicates statistically significant differences ($P \leq 0.005$) from the WT value of the respective day in culture.

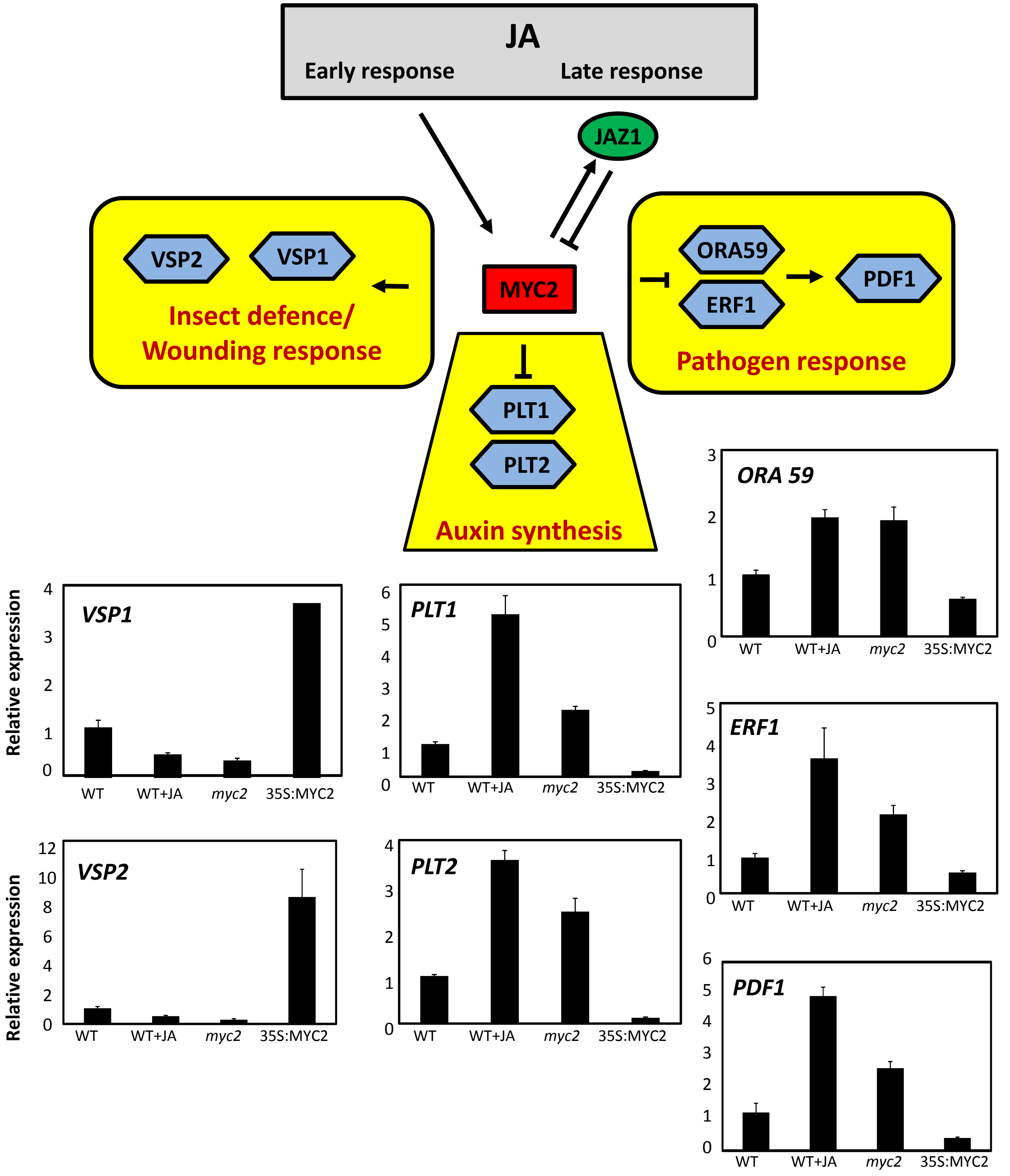

Supplemental Fig. 7. Diagram showing the effects of the “early” and “late” JA response on genes participating in insect defense/wounding response, pathogen response, and auxin synthesis. The expression of the genes participating in these responses (see the text for details) was measured at day 7 on induction medium in the WT line treated with JA and in lines with suppressed (*myc2*) or increased (35S:MYC2) levels of MYC2. Expression values are means of three biological replicated and are normalized to the value of WT set at 1.

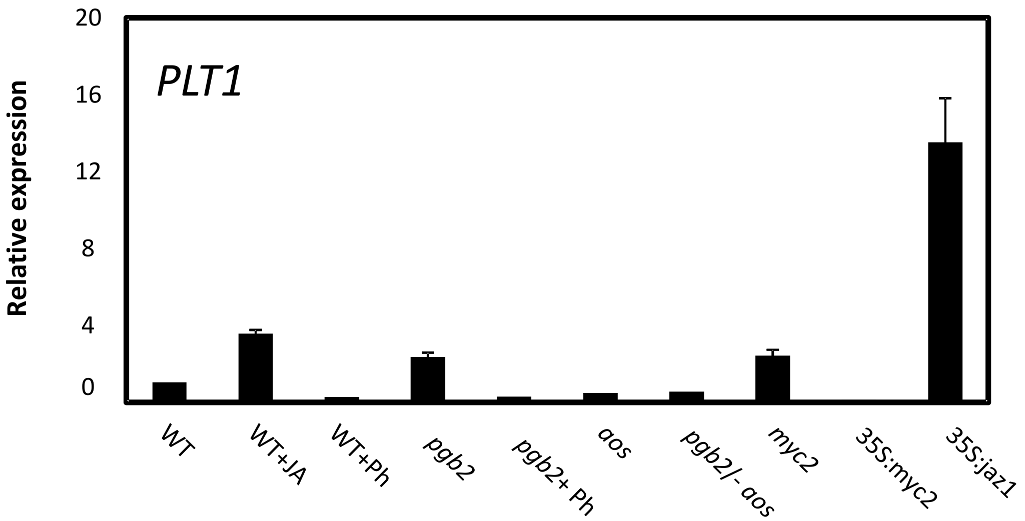

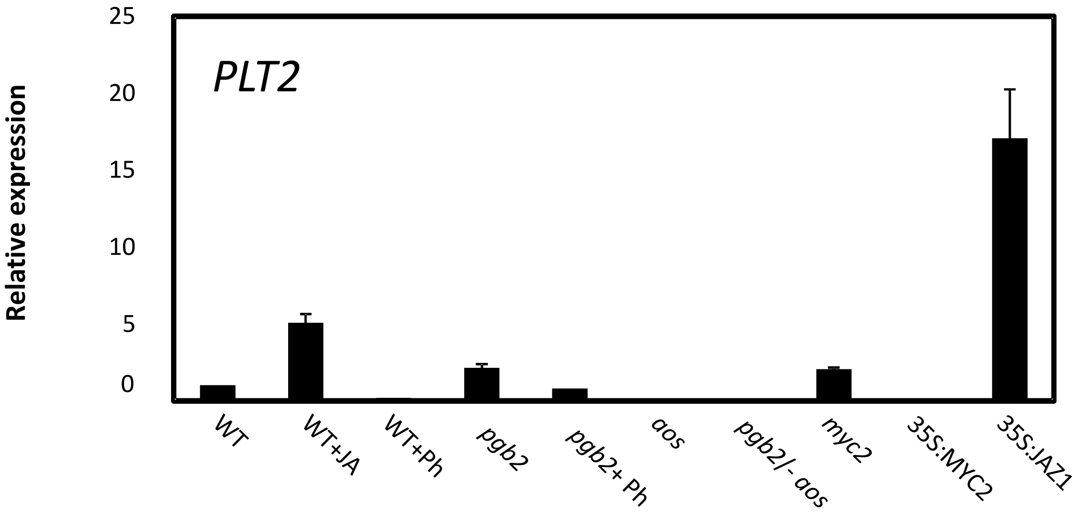

Supplemental Fig. 8. Expression levels of *PLETHORA 1* and *2* in day 7 explants. Gene expression was measured by (q)RT-PCR in the WT , *pgb2, aos*, myc2 and 35S:MYC2 lines. Values are means of three biological replicated and are normalized to the value of WT set at 1.

Supplemental Fig. 8

Supplemental Table 1. List of primers used for genotyping and for expression studies

**primers for genotyping**			
AOS-F	CGAGAAATTAACGGAGCTTCC	AT5G42650.1	ALLENE OXIDE SYNTHASE
AOS-R	CTAACCGGAGGCTACCGTATC	AT5G42650.1	ALLENE OXIDE SYNTHASE
LBb1-3	ATT TTG CCG ATT TCG GAA C		T-DNA insertion
PGB2-F	CTCAAAGCTCATGCTGTTAAAGTC	AT3G10520	hemoglobin-2
PGB2-R	TGTGTCAGCCACTACCACCT	AT3G10520	hemoglobin-2
dSmp-R	5-ACCGTCGACTACCTTTTTCTTGTAGTG-3		
**primers for RT-PCR**			
AtUBQ10-F	AACTTTGGTGGTTTGTGTTTTGG	AT4G05320	UBIQUITIN 10
AtUBQ10-R	TCGACTTGTCATTAGAAAGAAAGAGATAA	AT4G05320	UBIQUITIN 10
MYC2-F	ACCACGTCGAAGCAGAGAGACAAA	AT1G32640.1	JASMONATE INSENSITIVE 1
MYC2-R	TGTAAGCGATTGCGTCACCGAGTA	AT1G32640.1	JASMONATE INSENSITIVE 1
JAZ1-F	CCGGTTCTTGGAGAAGAGAAAG	AT1G19180.1	JASMONATE-ZIM-DOMAIN PROTEIN 1
JAZ1-R	CCTGTGGTTTGAGGGTTTGA	AT1G19180.1	JASMONATE-ZIM-DOMAIN PROTEIN 1
LOX2-F	ATGCTACGTCATGCTGGCTATGGA	AT3G45140	LIPOXYGENASE 2
LOX2-R	TGCCGCTATTATGTATGGCTCCGT	AT3G45140	LIPOXYGENASE 2
AOS-F	ACGACGCGGCGTTTAAAGTCAAAG	AT5G42650	ALLENE OXIDE SYNTHASE
AOS-R	ACGAATCTCTCCGGCACAAACTCA	AT5G42650	ALLENE OXIDE SYNTHASE
PDF1.2-F	GTTCTCTTTGCTGCTTTCGAC	AT5G44420	PLANT DEFENSIN 1.2
PDF1.2-R	GCAAACCCCTGACCATGT	AT5G44420	PLANT DEFENSIN 1.2
ASA1-F	ACAAGGATGCTAACAAACGGCGTG	AT1G19920	ATP SULFURYLASE ARABIDOPSIS 1
ASA1-R	TCTGGCACTCACAGTGTTCGTCTT	AT1G19920	ATP SULFURYLASE ARABIDOPSIS 1
Yuc4-F	CTAACGGATGGAAAGGAGAGAAG	AT4G32540.1	YUCCA 4
Yuc4-R	GCGATCTTAACGGCGTCATA	AT4G32540.1	YUCCA 4
AMI1-F	ATCTCGTCGGTGAAGCCAGAGTTT	AT1G08980	AMIDASE 1
AMI1-R	CCGAGCAAAGTTGAAAGAGCCGTT	AT1G08980	AMIDASE 1
PGB2-F	CTCAAAGCTCATGCTGTTAAAGTC	AT3G10520	Hemoglobin-2
PGB2-R	TGTGTCAGCCACTACCACCT	AT3G10520	Hemoglobin-2
